# NETMAGE: A human disease phenotype map generator for the network-based visualization of phenome-wide association study results

**DOI:** 10.1093/gigascience/giac002

**Published:** 2022-02-15

**Authors:** Vivek Sriram, Manu Shivakumar, Sang-Hyuk Jung, Yonghyun Nam, Lisa Bang, Anurag Verma, Seunggeun Lee, Eun Kyung Choe, Dokyoon Kim

**Affiliations:** Department of Biostatistics, Epidemiology & Informatics, Perelman School of Medicine, University of Pennsylvania, 19104 Philadelphia, Pennsylvania, USA; Department of Biostatistics, Epidemiology & Informatics, Perelman School of Medicine, University of Pennsylvania, 19104 Philadelphia, Pennsylvania, USA; Department of Biostatistics, Epidemiology & Informatics, Perelman School of Medicine, University of Pennsylvania, 19104 Philadelphia, Pennsylvania, USA; Department of Digital Health, SAIHST, Sungkyunkwan University, Samsung Medical Center, 06355 Seoul, Republic of Korea; Department of Biostatistics, Epidemiology & Informatics, Perelman School of Medicine, University of Pennsylvania, 19104 Philadelphia, Pennsylvania, USA; Ultragenyx Pharmaceutical, 94949 Novato, California, USA; Department of Medicine, Division of Translational Medicine and Human Genetics, Perelman School of Medicine, University of Pennsylvania, 19104 Philadelphia, Pennsylvania, USA; Graduate School of Data Science, Seoul National University, 08826 Seoul, Republic of Korea; Department of Biostatistics, Epidemiology & Informatics, Perelman School of Medicine, University of Pennsylvania, 19104 Philadelphia, Pennsylvania, USA; Department of Biostatistics, Epidemiology & Informatics, Perelman School of Medicine, University of Pennsylvania, 19104 Philadelphia, Pennsylvania, USA; Institute for Biomedical Informatics, University of Pennsylvania, 19104 Philadelphia, Pennsylvania, USA

**Keywords:** disease-disease network, PheWAS, comorbidity, disease complication, network medicine

## Abstract

**Background:**

Disease complications, the onset of secondary phenotypes given a primary condition, can exacerbate the long-term severity of outcomes. However, the exact cause of many of these cross-phenotype associations is still unknown. One potential reason is shared genetic etiology—common genetic drivers may lead to the onset of multiple phenotypes. Disease-disease networks (DDNs), where nodes represent diseases and edges represent associations between diseases, can provide an intuitive way of understanding the relationships between phenotypes. Using summary statistics from a phenome-wide association study (PheWAS), we can generate a corresponding DDN where edges represent shared genetic variants between diseases. Such a network can help us analyze genetic associations across the diseasome, the landscape of all human diseases, and identify potential genetic influences for disease complications.

**Results:**

To improve the ease of network-based analysis of shared genetic components across phenotypes, we developed the humaN disEase phenoType MAp GEnerator (NETMAGE), a web-based tool that produces interactive DDN visualizations from PheWAS summary statistics. Users can search the map by various attributes and select nodes to view related phenotypes, associated variants, and various network statistics. As a test case, we used NETMAGE to construct a network from UK BioBank (UKBB) PheWAS summary statistic data. Our map correctly displayed previously identified disease comorbidities from the UKBB and identified concentrations of hub diseases in the endocrine/metabolic and circulatory disease categories. By examining the associations between phenotypes in our map, we can identify potential genetic explanations for the relationships between diseases and better understand the underlying architecture of the human diseasome. Our tool thus provides researchers with a means to identify prospective genetic targets for drug design, using network medicine to contribute to the exploration of personalized medicine.

## Background

Disease complications refer to the onset of secondary phenotypes given a primary condition, while disease comorbidities refer to the co-occurrent presence or onset of multiple diseases [1]. Both forms of disease association can exacerbate the long-term severity of disease, and they vary drastically from phenotype to phenotype [1]. However, their causes are still not well understood. One potential reason for these cross-phenotype associations [2] could be shared genetic etiology—the same genetic drivers may cause multiple symptoms to appear over time [3].

Electronic health record (EHR)-linked biobanks capture both clinical and genetic information for large populations of patients [4]. These repositories contain both genetic and longitudinal phenotype data, including DNA samples, disease histories, laboratory measurements, lifestyle habits, and demographic information [4]. Given an EHR-linked biobank as input, a phenome-wide association study (PheWAS) can be used to calculate a multitude of associations between phenotypes and genetic variants, such as single-nucleotide polymorphisms (SNPs), in an unbiased manner [4].

**Figure 1:**
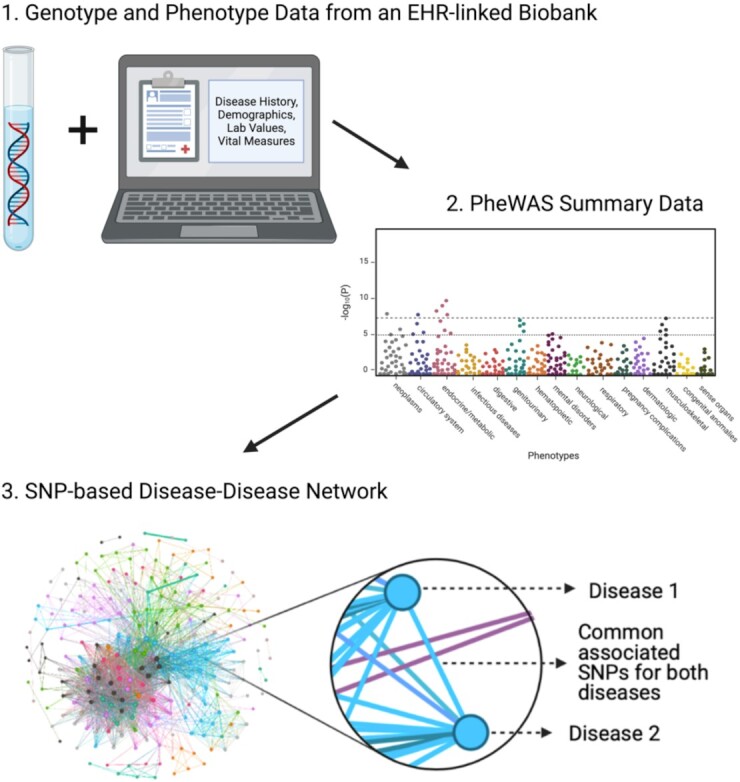
A depiction of the process for creating a SNP-based DDN. A PheWAS can be run on data from an EHR-linked biobank to calculate p-values of associations between a variety of single-nucleotide polymorphisms (SNPs) and phenotypes. The summary statistics from this PheWAS lend themselves to a DDN, where nodes represent diseases and edges represent common associated SNPs between diseases. Figure created with BioRender.com.

A holistic network-based view involving disorders across the diseasome will be required to translate these genetic correlations into an understanding of disease co-occurrences [5]. Disease-disease networks (DDNs), where nodes represent diseases and edges represent connections between diseases, can provide an intuitive way to understand the relationships between phenotypes [6, 7]. In particular, a DDN that uses its edges to represent variants can be generated as a proxy to highlight potential shared genetic influences for diseases (Figure 1). Analyzing the topology of these genetics-based DDNs can provide insight into how inherited factors may drive the onset of disease complications.

## Purpose of the Work

The network-based visualization of associations between variants and phenotypes can provide researchers and clinicians with a potential way to understand the genetic basis of disease interactions. In particular, the growth of available EHR-linked biobanks across institutions presents a trove of data that have yet to be mined from a “network medicine” perspective [5]. A variety of tools currently exist to depict PheWAS statistics, including PleioNet [8], ShinyGPA [9], PheGWAS [10], PheWeb [11], and PheWAS-ME [12] (Table 1). However, to our knowledge, none of these packages allows for the creation of interactive, searchable DDNs from user-provided PheWAS summary data.

**Table 1:** A comparison of NETMAGE to other toolkits that currently exist for the visualization of PheWAS summary statistics

Software Name	Allows users to upload desired PheWAS results for analysis	Allows for interactive investigation of cross-phenotype associations	Generates a network visualization of genetic associations between phenotypes	Allows users to search and create subsets of any produced networks by disease, by genetic variant, or by other network statistics
PleioNet		x	x	x
ShinyGPA	x	x		x
PheGWAS	x	x		N/A
PheWAS-Me	x	x		x
PheWeb	x	x		N/A
NETMAGE	x	x	x	x

N/A: not applicable.

**Figure 2:**
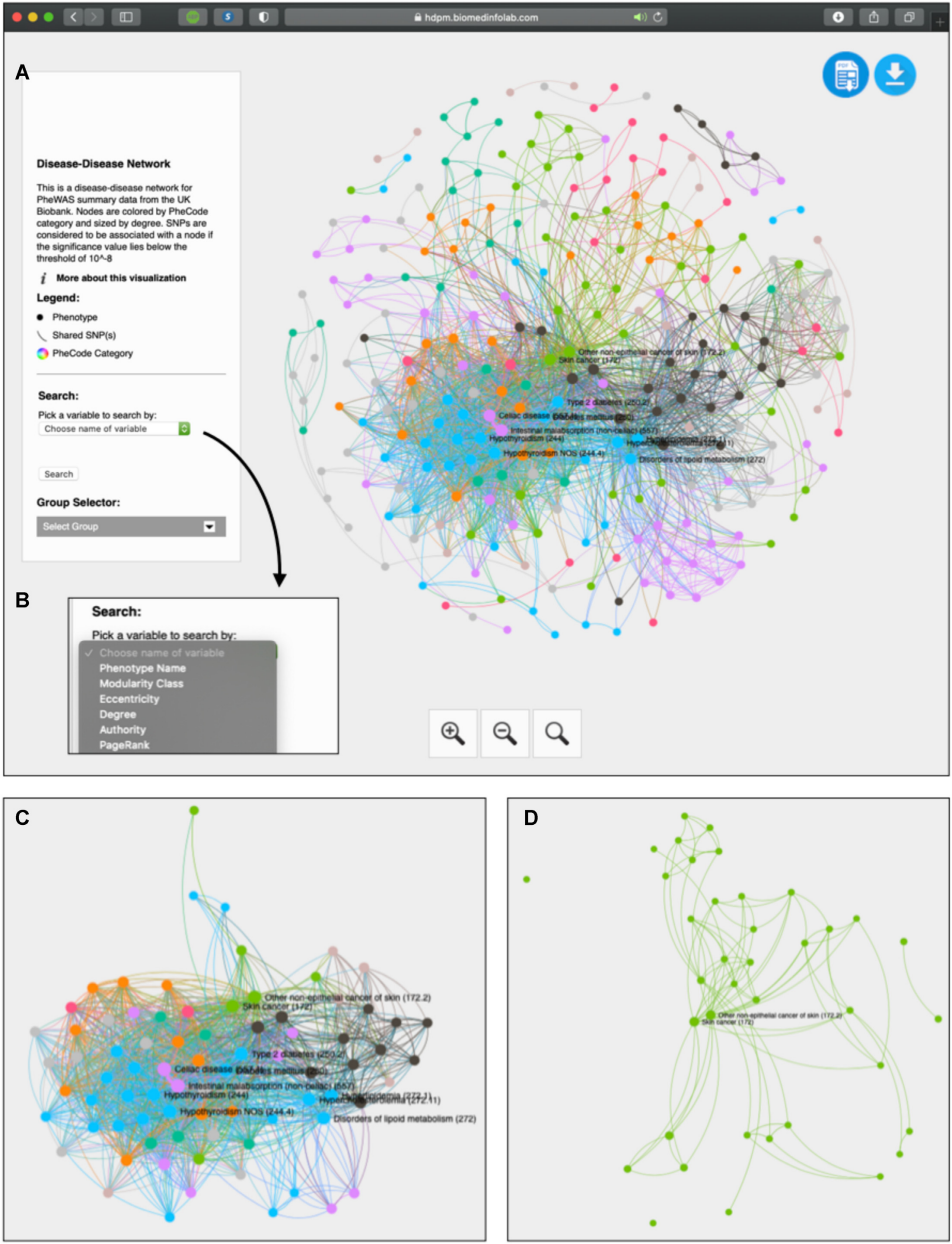
A depiction of the NETMAGE visualization tool. (A) The sidebar of the visualization gives a description of the map. It also includes a search dropdown and a group selector dropdown menu. (B) Variables are automatically read from the input data and included as options for search. (C) Clicking on a node reduces the displayed map to only the chosen node and its direct connections. Additionally, associated variants, connected phenotypes, and network statistics are presented to the right of the window when a node is selected. This graph corresponds to the subnetwork for type 2 diabetes. (D) All nodes within a single disease category can be visualized at once using the Group Selector. Here, we display all neoplasm phenotypes.

The humaN disEase phenoType MAp GEnerator (NETMAGE) addresses this need. NETMAGE (NETMAGE, RRID:SCR_021843) is a web-based tool that allows users to upload any PheWAS summary statistics and generate corresponding interactive networks. In particular, the resulting DDN is a projection of an undirected bipartite network of phenotypes and genetic variants, where nodes serve as diseases and edges serve as sets of common associated variants [6]. Users can filter their input data by p-value and by minor allele frequency (MAF) to manipulate the rarity and significance of variants being used to generate the network. Furthermore, they can select nodes within the DDN to view information such as connected phenotypes, shared variants, and network statistics (Fig. 2).

NETMAGE will serve as a step toward mass network-based analysis of PheWAS data. The interactive, graph-based representation of these summary statistics will help researchers visualize comorbidities as well as identify genetic variants that may potentially lead to the onset of disease complications. Furthermore, because NETMAGE facilitates the analysis of PheWAS data from individual EHR-linked biobanks, users can follow up with phenotypic data in their corresponding EHRs to evaluate the predictive ability of genetics-based DDNs with respect to disease co-occurrences. NETMAGE will allow us to gain a deeper understanding of the underlying genetic architecture of disease interaction.

## Implementation

We used Gephi (Gephi, RRID:SCR_004293) [13], an open-source network visualization software package, as well as InteractiveVis [14], a framework built over sigma.js [15] for the interactive visualization of geospatial data, as a base for the implementation of NETMAGE. These packages were extended to create a web interface for the generation of network visualizations. We implemented a web server backend to accept the files uploaded by the user and then parse and generate the network using the Gephi toolkit. We deployed the server on Amazon Web Services infrastructure, and it is available for use at the website [16]. We also enhanced the software to automatically parse all attributes provided in the input data and turn them into options for filtration and search. The NETMAGE pipeline works as follows:

1. Users upload their PheWAS summary statistic files to our website. Each row should correspond to a genetic variant, and the user can provide p-value and MAF information if they want to filter their data using NETMAGE. The data can be uploaded either as a single file where the phenotype name is included in each row or separate files where each file corresponds to a distinct phenotype.
2. NETMAGE converts PheWAS summary data into an intermediate disease_snpmap.netmage file. This file represents a dictionary of phenotype-to-variant mappings, where each phenotype serves as a key and each variant, p-value, MAF triplet serves as a value in a set. To create a DDN from the same data in the future, the user can simply upload the disease_snpmap.netmage file instead of re-uploading the original PheWAS data by using the “Upload netmage file” option.
3. The disease_snpmap.netmage file is converted into a corresponding node and edge map. Based upon the p-value and MAF thresholds provided by the user, phenotype-variant mappings will be filtered to provide a final file containing a list of relevant variants for each disease. This file is used to generate an edge map and a node map. The edge map establishes all links in the network—each row corresponds to an edge from a source to a target. Depending on the user's choice, the weight of the edge equals either the number of associated variants shared between the 2 phenotypes or the marginalized fraction of variants (the number of variants that constitute the edge divided by the union of the individual sets of variants for both phenotypes). In addition, the node map represents a list of all nodes in the network. Each row provides a distinct phenotype and a list of its associated variants. If input data have not already been pruned for linkage disequilibrium (LD), then users can provide an LD-mapping file that gives mappings between each variant to blocks of LD. NETMAGE will then clump SNPs according to their specified LD blocks, ensuring that associations that should be linking phenotypes together are present in the map. Users can also provide an input disease category mapping file so that each row of the node map now represents the disease and its category.
4. The node and edge maps are used to create a 2D mapping of the network. Through the Gephi and InteractiveVis frameworks, each disease is mapped to a 2D space to visualize the DDN. Within the NETMAGE web page, users can specify parameters including network layout, node size, and edge thickness to edit the aesthetics of the resulting graph.

Given a resulting network, NETMAGE offers the following features:

1. Node Selection: clicking on a node will highlight the node and all its first-degree neighbors. A variety of default attributes will be presented on the right side of the web page as part of an “Information Pane.” The user can also define other custom attributes, and these will be included in the Information Pane as well. If the user inputs data that include rsID-formatted SNPs, then NETMAGE will automatically hyperlink each SNP's ID to its corresponding dbSNP profile [17], allowing for further exploration of the variant's information. To aid with interpretation and visualization of disease associations, a hyperlink to a histogram of disease connections is also included in the Information Pane. For each phenotype, this histogram depicts first-degree disease neighbors sorted in order of the number of shared variants.
2. Search: users can search the map for relevant phenotypes based upon any attribute defined, such as phenotype name, phenotype ID, variant name, node degree, and other parameters. In particular, the “search by variant” option allows users to find shared genetic variants between diseases. The custom attributes provided by the user are also automatically incorporated into the search dropdown menu. Any categorical variables, such as disease name, disease category, or variant name, will include an autocompletion dropdown menu that dynamically updates as users type out their query terms.
3. Highlighting: groups of nodes within the same disease category can be highlighted to visualize associations within groups. These categories are established according to the user-provided input disease category file.

Key strengths of NETMAGE include the automated creation of DDNs from user input for the visualization of a multitude of datasets, searchability of DDNs by both phenotype and genetic variant, and interactivity with the nodes of the DDN. These aspects allow users to focus on specific genetic associations by visualizing subsets of the map. Generated networks can be interacted with online or downloaded in a static format. NETMAGE allows users to download an image of the network as a PDF file or download the data corresponding to the network, including the intermediate disease_snpmap.netmage file (providing a map of phenotypes to variants, including p-value and MAF information if given by the user), node and edge map files (providing all nodes in the network along with their attributes, as well as all edges in the network, respectively), and a final data.json file (providing the 2D mapping of the elements in network). The node and edge map files, as well as the data.json file, can all be visualized and edited locally within Gephi. The data.json file can also be directly hosted by users on any web server.

## Case Study

As a demonstration of the abilities of NETMAGE, we applied our software to SAIGE [18] -analyzed UK Biobank [19] (UKBB) PheWAS data. The current version of the DDN is hosted at the website [20]. These data corresponded to 1,403 binary phenotypes expressed in terms of PheCodes [21]. All 400,000 British individuals of European ancestry in the dataset were imputed using the Haplotype Reference Consortium panel, yielding 28 million imputed SNPs [11]. SAIGE [18], a generalized mixed model association test that uses the saddlepoint approximation to account for case-control imbalance, was used to generate summary statistics for each SNP, providing p-values of association between every SNP and every phenotype. This analysis was adjusted for genetic relatedness, sex, birth year, and the first 4 principal components [11]. All genomic positions are on GRCh37 [11]. Phenotypes that had a case count <200 were dropped to keep more relevant diseases, yielding a total of 1,075 traits for consideration. Data were also filtered to select significantly associated common variants, based upon the following thresholds: maximum p-value threshold [22] of 5 × 10^−8^, minimum MAF of 0.01, and LD-pruning through PLINK [23] length using the quality-controlled UKBB genetic data themselves as our reference panel, with an R^2^ of 0.2 and 250 kb for maximum search.

**Figure 3:**
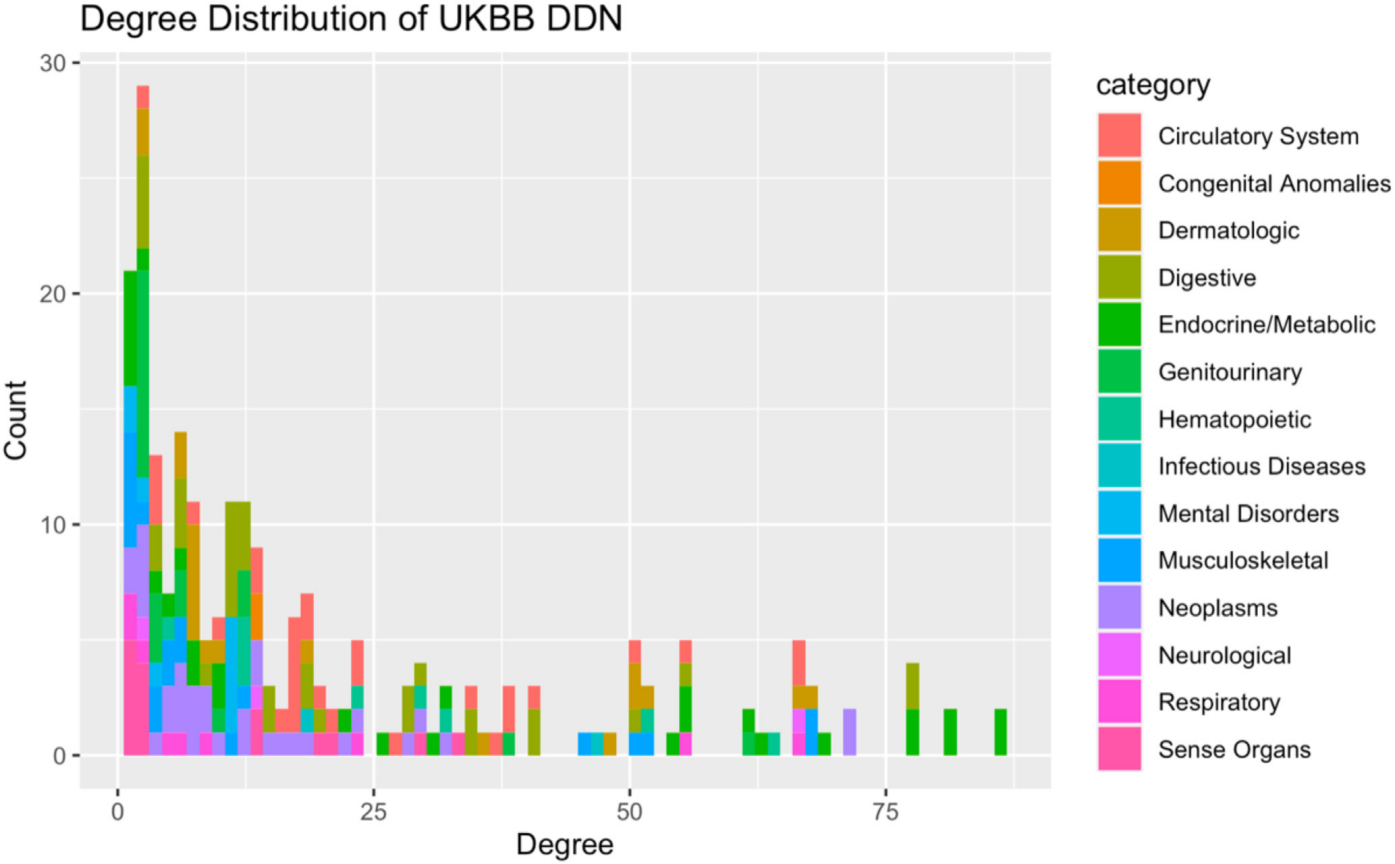
A histogram of degree distributions for the UKBB DDN. This distribution follows the power law, suggesting a scale-free property for the network. We also see that disease categories fail to follow specific trends based upon the degree of the disease.

Removing nodes with degree 0 after the previously described filtration steps yielded a final network of 232 nodes and 2,375 edges. Degrees of nodes ranged from 1 to 84. The mean degree was 20.47 and the mean weighted degree was 1,657.17. A total of 68% (158 of 232) nodes had lower degrees than the mean degree, implying a scale-free nature of the network (Fig. 3) [5]. Furthermore, the diameter of the network was 7 while the mean path length was 2.70, suggesting the small-world property for the network [5]. A total of 570 edges (24%) connect diseases of the same category while 1,805 edges (76%) connect diseases of different categories, indicating that the genetic associations we identified appeared mostly across disease classes. Modularity analysis yielded 18 different clusters, ranging from size 2 to 72. There was also extensive variation in terms of the disease categories present for each module, again suggesting that genetic associations with phenotypes are not specific to disease class. Finally, the mean clustering coefficient was 0.782, meaning that the network lacks extensive local clustering [5].

Degree, weighted degree, closeness centrality, betweenness centrality, and eigenvector centrality were all used to identify hub diseases in the DDN [5]. Diseases with the highest degree included hyperlipidemia (272.1), disorders of lipoid metabolism (272), type 2 diabetes (250.2), diabetes mellitus (250), and hypothyroidism (244.4). Diseases with the highest weighted degree included celiac disease (557.1), non-celiac intestinal malabsorption (557), hypothyroidism (244), type 1 diabetes (250.1), and psoriasis (696 and 696.4). Highest closeness centrality phenotypes included disorders of muscle, ligament, and fascia (728), fasciitis (728.7), and other retinal disorders (362), and highest betweenness centrality phenotypes included disorders of lipoid metabolism (272), hyperlipidemia (272.1), skin cancer (172), coronary atherosclerosis (411.4), hypertension (401), and essential hypertension (401.1). Finally, highest eigenvector centrality diseases included intestinal malabsorption and celiac disease (557 and 557.1), hypothyroidism (244.4 and 244), type 2 diabetes (250.2), type 1 diabetes (250.1), psoriasis (696), and rheumatoid arthritis and other inflammatory polyarthropathies (714.1 and 714). On the basis of these results, it appears that endocrine/metabolic and circulatory diseases seem to have the most influence in our DDN (Table 2).

**Table 2:** Hub phenotypes in the UKBB DDN

Phenotype	PheCode	Disease category
Skin cancer	172	Neoplasm
Diabetes mellitus	250	Endocrine/metabolic
**Hypothyroidism**	244	Endocrine/metabolic
**Hypothyroidism NOS**	244.4	Endocrine/metabolic
**Type 1 diabetes**	250.1	Endocrine/metabolic
**Type 2 diabetes**	250.2	Endocrine/metabolic
**Disorders of lipoid metabolism**	272	Endocrine/metabolic
**Hyperlipidemia**	272.1	Endocrine/metabolic
Other retinal disorders	362	Sense organs
Hypertension	401	Circulatory system
Essential hypertension	401.1	Circulatory system
Coronary atherosclerosis	411.4	Circulatory system
Non-celiac intestinal malabsorption	557	Digestive
**Celiac disease**	557.1	Digestive
**Psoriasis**	696	Dermatologic
Psoriasis NOS	696.4	Dermatologic
Other inflammatory polyarthropathies	714	Musculoskeletal
Rheumatoid arthritis	714.1	Musculoskeletal
Disorders of muscle, ligament, and fascia	728	Musculoskeletal
Fasciitis	728.7	Musculoskeletal

Centrality measures used to identify these phenotypes included degree, weighted degree, closeness centrality, betweenness centrality, and eigenvector centrality. Diseases marked in boldface appear multiple times as the most central nodes based upon our different network measures. Supplementary Table S1 provides the exact centrality measures that identified each phenotype to be a hub. NOS: not otherwise specified.

The DDN that we generated includes many disease connections identified in previous studies. In keeping with the DDN generated from the DiscovEHR biobank [7], our network identified connections among type 1 diabetes, rheumatoid arthritis, psoriasis, and multiple sclerosis. It also identified connections among hypothyroidism, type 2 diabetes, thyroid cancer, obesity, and rheumatoid arthritis. Furthermore, similar to the Disease Comorbidity Network [24] derived from hospitals across China, our DDN included edges between hypertension and hyperlipidemia, type 1 and type 2 diabetes, and diabetes mellitus. Finally, in keeping with a multimorbidity study performed on elderly patients in Tokyo [25], our DDN identified connections between hypertension, dyslipidemia, and coronary heart disease.

Finally, considering potential genetic associations between diseases, we find that our DDN displays relevant genetic associations between diseases, including rs544873’s association with pulmonary heart disease, phlebitis and thrombophlebitis, hemorrhoids, circulatory disease, and diverticulosis [26]; rs925488’s association with thyroid cancer, nontoxic nodular and multinodular goiter, and hypothyroidism [24]; and rs780094’s association with diabetes and lipid metabolism [27].

One potential issue in terms of the conclusions that can be drawn from our UKBB DDN is the use of “PheCodes” as a method of defining phenotypes. PheCodes are defined according to International Classification of Diseases (ICD) codes, but the accuracy of these codes for disease diagnosis is known to be questionable. Given such inaccuracies, users must be wary when treating PheCode or *ICD-*based diagnoses as a gold standard because doing so may lead to inaccurate conclusions. Another aspect of the use of PheCodes for phenotype definitions is their hierarchical nature. Digits that appear after decimal points correspond to subsets of phenotypes compared to the parent code that appears before the decimal. In our case study, the data that we make use of include mostly upper hierarchy phenotypes. More detailed hierarchical phenotypes are for the most part absent from our network. Users should be careful about including extensive hierarchical structure in their input data when generating DDNs through NETMAGE. Including phenotypes that are essentially identical to one another will introduce unnecessary nodes and edges in the network, in the process clouding more significant disease connections.

In terms of future work for this case study, it would be interesting to compare the edges in our DDN with known disease comorbidities. We can take disease occurrence data from an external EHR and evaluate φ-correlations between all pairs of phenotypes. Comparison of these co-occurrences to the genetic associations in our PheWAS may give us an indication whether the DDN is a reasonable representation of disease connections.

## Runtime Analysis

As a test of runtime for NETMAGE, we constructed DDNs from random subsets of the PheWAS data used to create the UKBB DDN and determined the time it took for each network to be generated. Five networks were each generated from collections of 50, 100, 250, 500, and 1,000 phenotypes. These DDNs were constructed in both the Fruchterman-Reingold and Force Atlas 2 layouts from Gephi [13], resulting in a total of 50 graphs for runtime analysis. The mean time to create a network seems to increase in *O*(*n*^2^) as the number of phenotypes increases (Table 3). This behavior makes sense because runtime depends on not only the number of phenotypes included in the input data but also the number of variants being tested. Indeed, if each additional phenotype added to the network will have multiple associated variants, then the inclusion of nodes will tend to exponentially increase the number of edges, assuming a low clustering coefficient in the network.

**Table 3:** Runtimes for DDN generation given input datasets with different numbers of phenotypes

	Server runtime to generate network after receiving HTTP request (sec)											
Phenotype count	Fruchterman-Reingold layout						Force Atlas 2 layout					
	1	2	3	4	5	Mean (SD)	1	2	3	4	5	Mean (SD)
50	3.07	2.34	2.86	2.31	2.76	2.67 (0.33)	2.46	2.48	2.93	2.43	3.00	2.66 (0.28)
100	3.26	3.49	4.29	3.61	3.52	3.63 (0.39)	3.43	4.14	4.37	4.62	3.58	4.03 (0.51)
250	6.60	5.20	6.77	6.62	5.56	6.15 (0.72)	6.74	5.31	6.36	6.92	5.90	6.25 (0.65)
500	11.21	11.85	12.53	10.94	9.91	11.29 (0.99)	11.68	12.04	12.49	11.21	9.33	11.35 (1.22)
1,000	28.27	28.77	30.19	27.01	29.52	28.75 (1.22)	29.37	28.35	29.84	27.23	30.23	29.00 (1.22)
UKBB DDN	48.60					N/A	39.43					N/A

These times measure how long it takes for the server to generate the network after the “submit” button has been clicked—in all instances, files have already been uploaded to the server. Upload speeds for files will vary depending on user bandwidth. Five different datasets were constructed for each count of phenotypes to evaluate runtime, and the mean and standard deviation of time for the 5 runs is also provided for each row. Finally, runtime for the full input UKBB case study is included in the last row of the table. N/A: not applicable.

## Discussion and Conclusions

NETMAGE is a toolkit for the network-based interactive visualization of PheWAS summary data. The goal of this software is to improve the ease of visualization of genetic associations across diseases and to facilitate large-scale genetic analysis of the human diseasome. While the UKBB data used for our case study consisted of entirely binary phenotypes, NETMAGE is also applicable to quantitative traits. Indeed, in such a situation, the continuous value of the quantitative phenotype, such as a laboratory test measure like A1C level, is used as the outcome variable in the PheWAS. This process provides a more detailed degree of association between the severity of the trait and genetic variants, as compared to the identification of associations between a presence or absence of the trait with variants.

A key point to note regarding NETMAGE is that the output DDNs will provide only as much information as the input data. Indeed, NETMAGE is an exploratory tool intended to help visualize connections between diseases. Including summary PheWAS data that provide insight into the statistical associations between phenotypes will yield an associative map but will tell us nothing about causality. Associations identified through PheWAS are often spurious, so any sort of analyses performed on these data must take this information into consideration. Nevertheless, these kinds of associative visualizations are still useful for the study of disease and may help identify connections between phenotypes and genetic variants, generate new hypotheses, and suggest future experiments that can be conducted. For a visualization that gives stronger insight into the causal connections between traits, one could potentially input the results of a Mendelian randomization experiment.

Several future directions exist for NETMAGE. First is the inclusion of directionality in the network—as of now, DDNs produced by NETMAGE give no indication regarding the direction of association between phenotypes. Using β-values for the association between phenotypes and genetic variants would be a useful inclusion, aiding in clinical interpretation of the network. We will also allow for the concurrent selection of multiple nodes within the DDN. The current NETMAGE user interface allows only 1 node to be selected at a time. The ability to select multiple nodes will allow clinicians to quickly identify whether 2 phenotypes are associated in the network. We also hope to enhance NETMAGE to allow for the construction of gene-based DDNs from variant-based data by including variant-to-gene mapping as a part of the website. Finally, we will allow users to create variant-variant networks instead of disease-disease networks, which depict the connections between genetic variants (e.g., SNPs) based upon associations with phenotypes.

Ultimately, NETMAGE will give researchers and clinicians insight into the underlying genetic architecture of disease complications. The impact of our work will be a tool that allows for the potential identification of new gene targets that can be investigated in follow-up studies of pleiotropy and drug discovery. We hope that this software will contribute to new potential discoveries in personalized medicine and that it helps facilitate the advancement of network medicine studies into the genetics of disease co-occurrences.

## Availability of Supporting Source Code and Requirements

- Project name: NETMAGE
- Project home page: https://hdpm.biomedinfolab.com/netmage/
- Source code: https://github.com/dokyoonkimlab/netmage
- RRID: SCR_021843
- biotools:netmage
- Operating system: Platform independent
- Programming language: Python, HTML, JavaScript
- Other requirements: None

## Data Availability

Supporting data and materials are available in the *GigaDB* database [28].

## Additional Files

**Table S1**. A table of phenotypes with the highest centrality measures in the UKBB DDN. Diseases marked in boldface appear multiple times as the most central nodes based upon our different network measures.

giac002_GIGA-D-21-00220_Original_Submission

giac002_GIGA-D-21-00220_Revision_1

giac002_Response_to_Reviewer_Comments_Original_Submission

giac002_Reviewer_1_Report_Original_Submission Sarah Gagliano Taliun, PhD -- 8/3/2021 Reviewed

giac002_Reviewer_1_Report_Revision_1 Sarah Gagliano Taliun, PhD -- 11/30/2021 Reviewed

giac002_Reviewer_2_Report_Original_Submission Dongjun Chung -- 8/15/2021 Reviewed

giac002_Reviewer_2_Report_Revision_1 Dongjun Chung -- 12/14/2021 Reviewed

giac002_Reviewer_3_Report_Original_Submission Yaomin Xu -- 9/1/2021 Reviewed

giac002_Supplemental_File

## Abbreviations

DDN: disease-disease network; EHR: electronic health record; LD: linkage disequilibrium; MAF: minor allele frequency; NETMAGE: humaN disEase phenoType MAp GEnerator; PheWAS: phenome-wide association study; SNP: single-nucleotide polymorphism; UKBB: UK BioBank.

## Funding

This work has been supported by the National Institute of General Medical Sciences (NIGMS) R01 GM138597 and S10OD023495.

## Conflict of Interest

The authors declare that they have no competing interests.

## Author Contributions

VS, MS, EKC, and DK were involved in designing and conceptualizing the study. EKC and DK supervised the study. VS, MS, and SHJ performed data organization. VS, MS, SHJ, YN, and AV contributed to data analysis. VS and MS wrote the manuscript. All authors revised and approved the final manuscript.
