# Supplementary material for: NETMAGE: A human disease phenotype map generator for the network-based visualization of phenome-wide association study results
Source: Gigascience. 2022 Feb 15;11:giac002. doi: 10.1093/gigascience/giac002 (PMC8848314; doi:10.1093/gigascience/giac002)
Supplement: giac002_GIGA-D-21-00220_Original_Submission [file giac002_giga-d-21-00220_original_submission.pdf]

GigaScience

NETMAGE: A Human Disease Phenotype Map Generator for the Network-based Visualization of PheWAS Results

--Manuscript Draft--

**Manuscript Number:**	GIGA-D-21-00220
**Full Title:**	NETMAGE: A Human Disease Phenotype Map Generator for the Network-based Visualization of PheWAS Results
**Article Type:**	Technical Note
**Funding Information:**	national institute of general medical sciences (R01 GM138597) — Dr. Dokyoon Kim
**Abstract:**	Background<br>Disease complications, the onset of secondary phenotypes given a primary condition, can exacerbate the long-term severity of outcomes. However, the exact cause of many of these cross-phenotype associations is still unknown. One potential reason is shared genetic etiology – common genetic drivers may lead to the onset of multiple phenotypes. A holistic, network-based view incorporating knowledge of other diseases and genetic associations will be required to uncover the exact basis of disease complications. Disease-disease networks (DDNs), where nodes represent diseases and edges represent associations between diseases, can provide an intuitive way of understanding the relationships between phenotypes. Using summary statistics from a phenome-wide association study (PheWAS), we can generate a corresponding DDN where edges represent shared single-nucleotide polymorphisms (SNPs) between diseases. Such a network can help us analyze genetic associations across the diseasome, the landscape of all human diseases, and identify potential genetic influences for disease complications.<br>Results<br>To improve the ease of network-based analysis of shared genetic components across phenotypes, we developed the humaN disEase phenoType MAp GEnerator (NETMAGE), a web-based tool that produces interactive DDN visualizations from PheWAS summary statistics. Users can search the map by various attributes and select nodes to view related phenotypes, associated SNPs, and various network statistics. As a test case, we used NETMAGE to construct a network from UK BioBank (UKBB) PheWAS summary statistic data. Our map correctly displayed previously identified disease comorbidities from the UKBB and identified concentrations of hub diseases in the endocrine/metabolic and circulatory disease categories. By examining the associations between phenotypes in our map, we can identify potential genetic explanations for the relationships between diseases and better understand the underlying architecture of the human diseasome. Our tool thus provides researchers with a means to identify prospective genetic targets for drug design, using network medicine to contribute to the exploration of personalized medicine.<br>Availability: Our service runs at https://hdpm.biomedinfolab.com. Source code can be downloaded from https://github.com/dokyoonkimlab/netmage.
**Corresponding Author:**	Dokyoon Kim<br>University of Pennsylvania<br>Philadelphia, PA UNITED STATES
**Corresponding Author Secondary Information:**	
**Corresponding Author's Institution:**	University of Pennsylvania
**Corresponding Author's Secondary Institution:**	
**First Author:**	Vivek Sriram
**First Author Secondary Information:**	
**Order of Authors:**	Vivek Sriram

Powered by Editorial Manager® and ProduXion Manager® from Aries Systems Corporation

	Manu Shivakumar
	Sang-Hyuk Jung
	Yonghyun Nam
	Lisa Bang
	Anurag Verma
	Seunggeun Lee
	Eun Kyung Choe
	Dokyoon Kim
**Order of Authors Secondary Information:**	
**Additional Information:**	
**Question**	**Response**
Are you submitting this manuscript to a special series or article collection?	No
**Experimental design and statistics**<br><br>Full details of the experimental design and statistical methods used should be given in the Methods section, as detailed in our Minimum Standards Reporting Checklist. Information essential to interpreting the data presented should be made available in the figure legends.<br><br>Have you included all the information requested in your manuscript?	Yes
**Resources**<br><br>A description of all resources used, including antibodies, cell lines, animals and software tools, with enough information to allow them to be uniquely identified, should be included in the Methods section. Authors are strongly encouraged to cite Research Resource Identifiers (RRIDs) for antibodies, model organisms and tools, where possible.<br><br>Have you included the information requested as detailed in our Minimum Standards Reporting Checklist?	Yes
**Availability of data and materials**	Yes

All datasets and code on which the conclusions of the paper rely must be either included in your submission or deposited in publicly available repositories (where available and ethically appropriate), referencing such data using a unique identifier in the references and in the “Availability of Data and Materials” section of your manuscript.<br><br>Have you have met the above requirement as detailed in our Minimum Standards Reporting Checklist?	

Manuscript

Click here to access/download;Manuscript;NETMAGE_outline_gigaScience_0

NETMAGE: A Human Disease Phenotype Map Generator for the Network-based Visualization of PheWAS Results

Vivek Sriram[1§] – viveksrm@pennmedicine.upenn.edu
Manu Shivakumar[1§] – Manu.Shivakumar@pennmedicine.upenn.edu
Sang-Hyuk Jung[1,2] – normal.hyuk@gmail.com
Yonghyun Nam[1] – yonghyun.nam@pennmedicine.upenn.edu
Lisa Bang[3] – lisagbang@gmail.com
Anurag Verma[4] – anurag.verma@pennmedicine.upenn.edu
Seunggeun Lee[5] – leeshawn@umich.edu
Eun Kyung Choe[1] – choe523@gmail.com
Dokyoon Kim[1*] – dokyoon.kim@pennmedicine.upenn.edu

[1] Department of Biostatistics, Epidemiology & Informatics, Perelman School of Medicine, University of Pennsylvania, Philadelphia, PA 19104, USA

[2] Samsung Advanced Institute for Health Sciences and Technology (SAIHST), Sungkyunkwan University, Samsung Medical Center, Seoul, Republic of Korea

[3] Ultragenyx Pharmaceutical, Novato, CA 94949, USA

[4] Department of Medicine, Division of Translational Medicine and Human Genetics, Perelman School of Medicine, University of Pennsylvania, Philadelphia, PA 19104, USA

[5] Graduate School of Data Science, Seoul National University, Seoul, Republic of Korea

Corresponding Author: Dokyoon Kim

Abstract

Background

Disease complications, the onset of secondary phenotypes given a primary condition, can exacerbate the long-term severity of outcomes. However, the exact cause of many of these cross-phenotype associations is still unknown. One potential reason is shared genetic etiology – common genetic drivers may lead to the onset of multiple phenotypes. A holistic, network-based view incorporating knowledge of other diseases and genetic associations will be required to uncover the exact basis of disease complications. Disease-disease networks (DDNs), where nodes represent diseases and edges represent associations between diseases, can provide an intuitive way of understanding the relationships between phenotypes. Using summary statistics from a phenome-wide association study (PheWAS), we can generate a corresponding DDN where edges represent shared single-nucleotide polymorphisms (SNPs) between diseases. Such a network can help us analyze genetic associations across the diseasome, the landscape of all human diseases, and identify potential genetic influences for disease complications.

Results

To improve the ease of network-based analysis of shared genetic components across phenotypes, we developed the humaN disEase phenoType MAp GEnerator (NETMAGE), a web-based tool that produces interactive DDN visualizations from PheWAS summary statistics. Users can search the map by various attributes and select nodes to view related phenotypes, associated SNPs, and various network statistics. As a test case, we used NETMAGE to construct a network from UK BioBank (UKBB) PheWAS summary statistic data. Our map correctly displayed previously identified disease comorbidities from the UKBB and identified concentrations of hub diseases in the endocrine/metabolic and circulatory disease categories. By examining the associations between phenotypes in our map, we can identify potential genetic explanations for the relationships between diseases and better understand the underlying architecture of the human diseasome. Our tool thus provides researchers with a means to identify prospective genetic targets for drug design, using network medicine to contribute to the exploration of personalized medicine.

Availability: Our service runs at https://hdpm.biomedinfolab.com. Source code can be downloaded from https://github.com/dokyoonkimlab/netmage.

Contact: dokyoon.kim@pennmedicine.upenn.edu

Keywords

Disease-disease network; PheWAS; comorbidity; disease complication; network medicine

Findings

Background

Disease complications refer to the onset of secondary phenotypes given a primary condition, while disease comorbidities refer to the co-occurrent presence or onset of multiple diseases.[1] Both forms of disease association can exacerbate the long-term severity of disease, and they vary drastically from phenotype to phenotype.[1] However, their causes are still not well understood. One potential reason for these cross-phenotype associations[2] could be shared genetic etiology – the same genetic drivers may cause multiple symptoms to appear over time.[3]

Electronic health record (EHR)-linked biobanks capture both clinical and genetic information for large populations of patients.[4] These repositories contain both genetic and longitudinal phenotype data, including DNA samples, disease histories, laboratory measurements, lifestyle habits, and demographic information.[4] Given an EHR-linked biobank as input, a phenome-wide association study (PheWAS) can be used to calculate a multitude of associations between phenotypes and single-nucleotide polymorphisms (SNPs) in an unbiased manner.[4]

A holistic network-based view involving disorders across the diseasome will be required to translate these genetic correlations into an understanding of disease co-occurrences.[5] Disease-disease networks (DDNs), where nodes represent diseases and edges represent connections between diseases, can provide an intuitive way to understand the relationships between phenotypes.[6,7] In particular, a DDN that uses its edges to represent SNPs can be generated as a proxy to highlight potential shared genetic influences for diseases. Analyzing the topology of these SNP-based DDNs can provide insight into how genetic influences may drive the onset of disease complications.

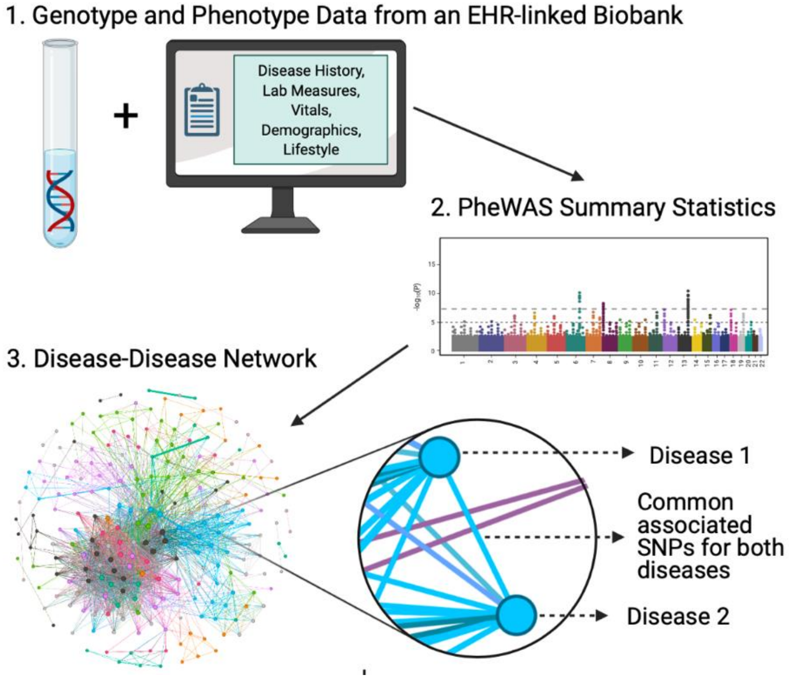

Figure 1. A depiction of the process for creating an SNP-based DDN. A PheWAS can be run on data from an EHR-linked biobank to calculate p-values of associations between a variety of genetic variants and phenotypes. The summary statistics from this PheWAS lend themselves to a DDN, where nodes represent diseases and edges represent common associated SNPs between diseases. Created with BioRender.com.

Purpose of the work

The network-based visualization of associations between SNPs and phenotypes can provide researchers and clinicians with a potential way to understand the genetic basis of disease interactions. In particular, the growth of available EHR-linked biobanks across institutions presents a trove of data that have yet to be mined from a "network medicine" perspective.[5] A variety of tools currently exist to depict PheWAS statistics, including PleioNet[8], ShinyGPA[9], PheGWAS[10], PheWeb[11], and PheWAS-ME[12] (Table 1). However, to the best of our knowledge, none of these packages allows for the creation of interactive, searchable DDNs from user-provided PheWAS summary data.

Table 1. A comparison of NETMAGE to other toolkits that currently exist for the visualization of PheWAS summary statistics.

Software Name	***Allows users to upload desired PheWAS results for analysis***	***Allows for interactive investigation of cross-phenotype associations***	***Generates a network visualization of genetic associations between phenotypes***	***Allows users to search and create subsets of any produced networks by disease, by SNP, or by other network statistics***
**PleioNet**		x	x	x
**ShinyGPA**	x	x		x
**PheGWAS**	x	x		N/A
**PheWAS-Me**	x	x		x
**PheWeb**	x	x		N/A
**NETMAGE**	x	x	x	x

The humaN disEase phenoType MAp GEnerator (NETMAGE) addresses this need. NETMAGE is a web-based tool that allows users to upload any PheWAS summary statistics and generate corresponding interactive networks. In particular, the resulting DDN is a projection of an undirected bipartite network of phenotypes and SNPs, where nodes serve as diseases and edges serve as sets of common associated SNPs.[6] Users can filter their input data by p-value and by minor allele frequency (MAF) to manipulate the rarity and significance of SNPs being used to generate the network. Furthermore, they can select nodes within the DDN to view information such as connected phenotypes, shared SNPs, and network statistics (Figure 2).

NETMAGE will serve as a step toward mass network-based analysis of PheWAS data. The interactive, graph-based representation of these summary statistics will help researchers visualize comorbidities as well as identify genetic variants that may potentially lead to the onset of disease complications. Furthermore, because NETMAGE facilitates the analysis of PheWAS data from individual EHR-linked biobanks, users can follow up with phenotypic data in their corresponding EHRs to evaluate the predictive ability of SNP-based DDNs with respect to disease co-occurrences. NETMAGE will allow us to gain a deeper understanding of the underlying genetic architecture of disease interaction.

Implementation

We used Gephi[13], an open-source network visualization software package, as well as InteractiveVis[14], a framework built over sigma.js[15] for the interactive visualization of geospatial data, as a base for the implementation of NETMAGE. These packages were extended to create a web interface for the generation of network visualizations. We implemented a web server backend to accept the files uploaded by the user and then parse and generate the network using the Gephi toolkit. We deployed the server on Amazon Web Service (AWS) infrastructure, and it is available for use at https://hdpm.biomedinfolab.com/netmage/. We also enhanced the software to automatically parse all attributes provided in the input data and turn them into options for filtration and search. The NETMAGE pipeline works as follows:

1. **Users upload their PheWAS summary statistic files to our website**. Each row should correspond to an SNP, and the user can provide p-value and MAF information if they want to filter their data using NETMAGE. The data can be uploaded either as a single file where the phenotype name is included in each row or separate files where each file corresponds to a distinct phenotype.
2. **NETMAGE converts PheWAS summary data into an intermediate *disease_snpmap.netmage* file.** This file represents a dictionary of phenotype-to-SNP mappings, where each phenotype serves as a key and each SNP, p-value, MAF triplet serves as a value in a set. To create a DDN from the same data in the future, the user can simply upload the *disease_snpmap.netmage* file instead of re-uploading the original PheWAS data by using the "Upload netmage file" option.
3. **The *disease_snpmap.netmage* file is converted into a corresponding node and edge map.** Based upon the p-value and MAF thresholds provided by the user, phenotype-SNP mappings will be filtered to provide a final file containing a list of relevant SNPs for each disease. This file is used to generate an edge map and a node map. The edge map establishes all links in the network – each row corresponds to an edge from a source to a target. The weight of the edge is equal to the number of associated SNPs shared between the two phenotypes. In addition, the node map represents a list of all nodes in the network. Each row provides a distinct phenotype and a list of its associated SNPs. Users can provide an input disease category mapping file so that each row of the node map now represents the disease and its category.
4. **The node and edge maps are used to create a two-dimensional mapping of the network.** Through the Gephi and InteractiveVis frameworks, each disease is mapped to a two-dimensional space to visualize the DDN. Within the NETMAGE webpage, users can specify parameters including network layout, node size, and edge thickness to edit the aesthetics of the resulting graph.

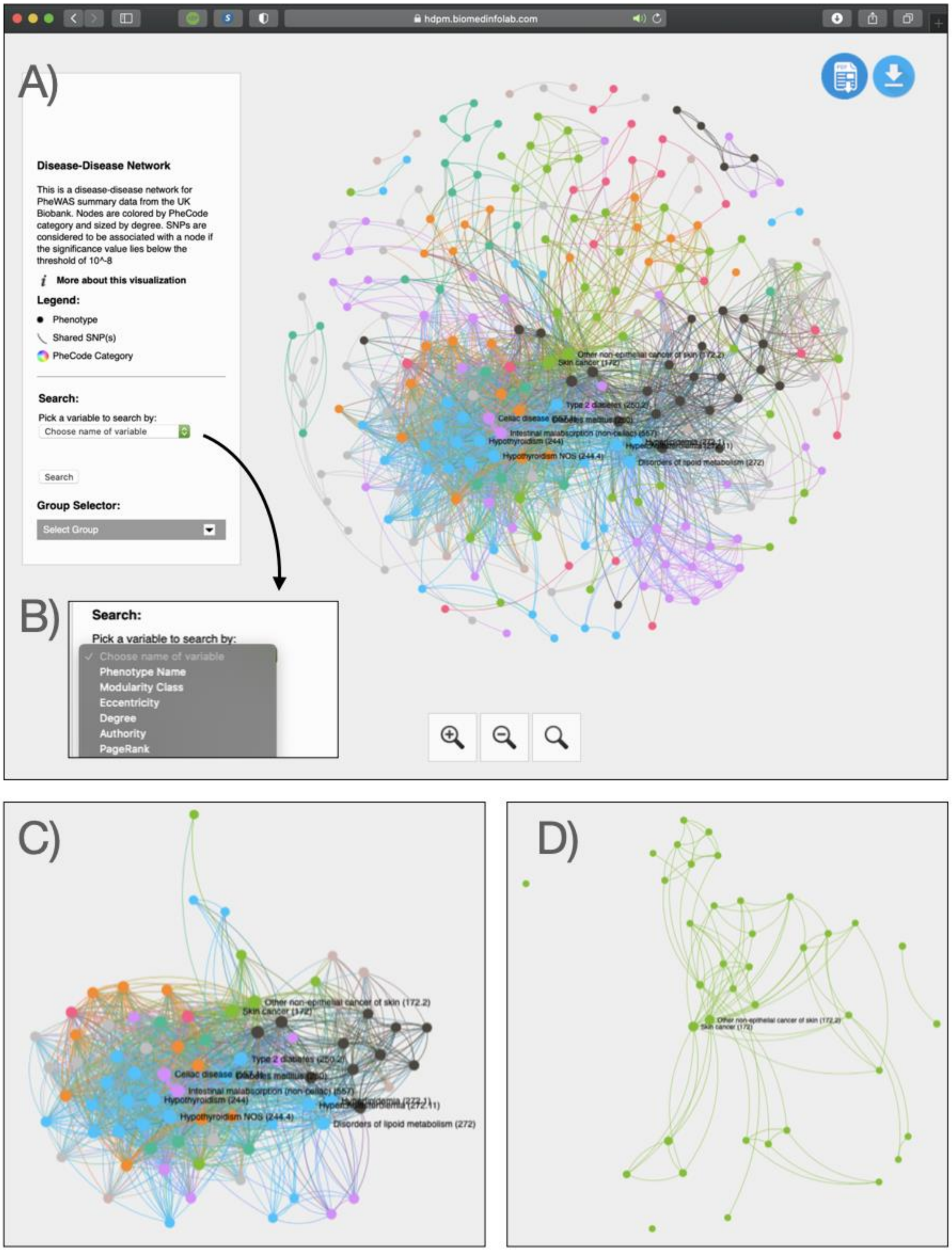

Figure 2. A depiction of the NETMAGE visualization tool. (A) The sidebar of the visualization gives a description of the map. It also includes a search dropdown and a group selector dropdown menu. (B) Variables are automatically read from the input data and included as options for search. (C) Clicking on a node reduces the displayed map to only the chosen node and its direct connections. Additionally, associated SNPs, connected phenotypes, and network statistics are presented to the right of the window when a node is selected. This graph corresponds to the subnetwork for type 2 diabetes (D) All nodes within a single disease category can be visualized at once using the Group Selector. Here, we display all neoplasm phenotypes.

Given a resulting network, NETMAGE offers the following features:

- **Node Selection**: clicking on a node will highlight the node and all of its first-degree neighbors. A variety of default attributes will be presented on the right side of the webpage. The user can also define other custom attributes.
- **Search**: users can search the map for relevant phenotypes based upon any attribute defined, such as phenotype name, phenotype ID, SNP ID, node degree, and other parameters. In particular, the “search by SNP” option allows users to find shared SNPs between diseases. The custom attributes provided by the user are also automatically incorporated into the search dropdown menu.
- **Highlighting**: groups of nodes within the same disease category can be highlighted to visualize associations within groups. These categories are established according to the user-provided input disease category file.

Key strengths of NETMAGE include the automated creation of DDNs from user input for the visualization of a multitude of datasets, searchability of DDNs by both phenotype and SNP, and interactivity with the nodes of the DDN. These aspects allow users to focus on specific genetic associations by visualizing subsets of the map. Generated networks can be interacted with online or downloaded in a static format. NETMAGE allows users to download an image of the network as a PDF file or download the data corresponding to the network, including the intermediate *disease_snpmap.netmage* file (providing a map of phenotypes to SNPs, including p-value and MAF information if given by the user), node and edge map files (providing all nodes in the network along with their attributes, as well as all edges in the network respectively), and a final *data.json* file (providing the two-dimensional mapping of the elements in network). The node and edge map files, as well as the *data.json* file, can all be visualized and edited locally within Gephi. The *data.json* file can also be directly hosted by users on any web server.

Case Study

As a demonstration of the abilities of NETMAGE, we applied our software to SAIGE[16]-analyzed UK Biobank[17] (UKBB) PheWAS data. The DDN is hosted at https://hdpm.biomedinfolab.com/ddn/ukbb. These data corresponded to 1,403 binary phenotypes expressed in terms of PheCodes[18] and 28 million imputed genetic variants for 400,000 British individuals of European ancestry. SAIGE[16] was used to generate summary statistics for each variant, providing p-values of association between every variant and every phenotype. Data were also filtered in order to select significantly associated common variants, based upon the following thresholds: maximum p-value threshold[19] of $5x10^{-8}$, minimum MAF of 0.01, minimum case count of 200, and LD-pruning through PLINK[20] with an R^2 of 0.2 and 250 kilobases for maximum search length.

The final network included 232 nodes and 2375 edges. Degrees of nodes ranged from 1 to 84. The average degree was 20.47 and the average weighted degree was 1657.17. 68% (158/232) nodes had lower degrees than the average degree, implying a scale-free nature of the network (Figure 3).[5] Furthermore, the diameter of the network was 7 while the average path length was 2.70, suggesting the small-world property for the network.[5] 570 edges (24%) connect diseases of the same category while 1,805 edges (76%) connect diseases of different categories, indicating that the genetic associations we identified appeared mostly across disease classes. Modularity analysis yielded 18 different clusters, ranging from size 2 to 72. There was also extensive variation in terms of the disease categories present for each module, again suggesting that genetic associations with phenotypes are not specific to disease class. Finally, the average clustering coefficient was 0.782, meaning that the network lacks extensive local clustering.[5]

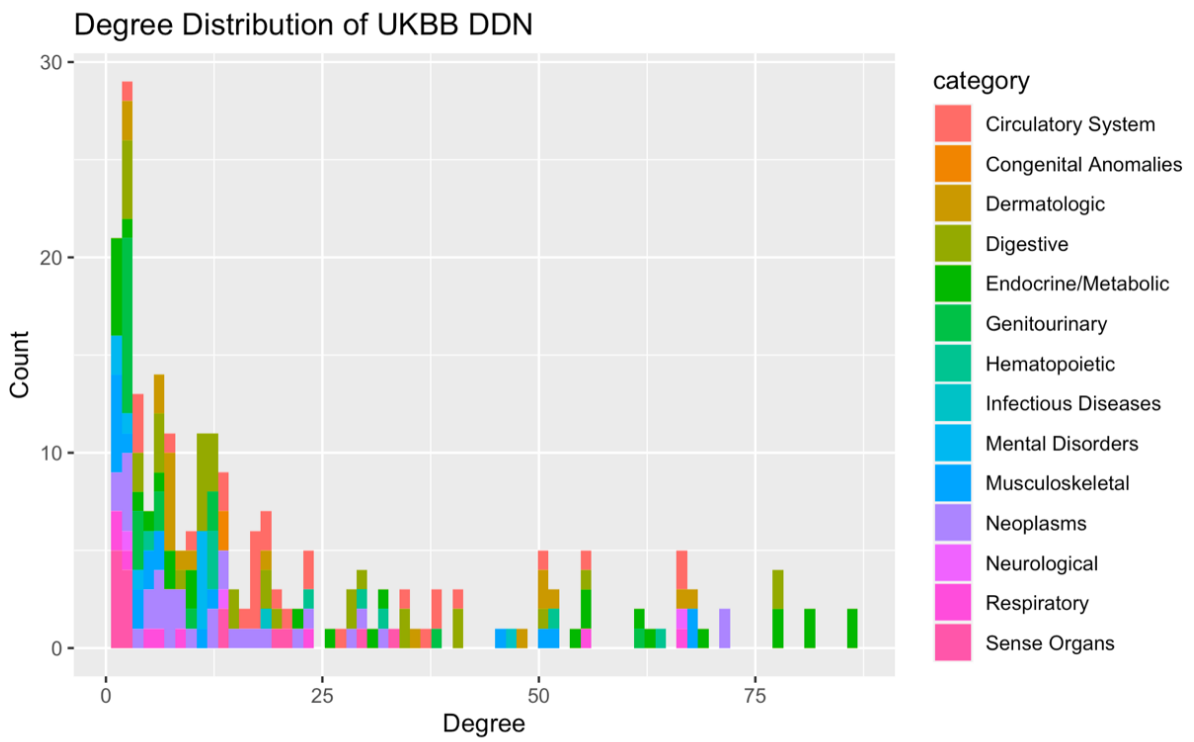

Figure 3. A histogram of degree distributions for the UKBB DDN. This distribution follows the power law, suggesting a scale-free property for the network. We also see that disease categories fail to follow specific trends based upon the degree of the disease.

Degree, weighted degree, closeness centrality, betweenness centrality, and eigenvector centrality were all used to identify hub diseases in the DDN.[5] Diseases with the highest degree included hyperlipidemia (272.1), disorders of lipoid metabolism (272), type 2 diabetes (250.2), diabetes mellitus (250), and hypothyroidism (244.4). Diseases with the highest weighted degree included celiac disease (557.1), non-celiac intestinal malabsorption (557), hypothyroidism (244), type 1 diabetes (250.1), and psoriasis (696 and 696.4). Highest closeness centrality phenotypes included disorders of muscle, ligament, and fascia (728), fasciitis (728.7), and other retinal disorders (362), and highest betweenness centrality phenotypes included disorders of lipoid metabolism (272), hyperlipidemia (272.1), skin cancer (172), coronary atherosclerosis (411.4), hypertension (401), and essential hypertension (401.1). Finally, highest eigenvector centrality diseases included intestinal malabsorption and celiac disease (557 and 557.1), hypothyroidism (244.4 and 244), type 2 diabetes (250.2), type 1 diabetes (250.1), psoriasis (696), and rheumatoid arthritis and other inflammatory polyarthropathies (714.1 and 714). Based upon these results, it appears that endocrine/metabolic and circulatory diseases seem to have the most influence in our DDN (Table 2).

Table 2. A table of phenotypes with the highest centrality measures in the UKBB DDN. Diseases marked in bold appear multiple times as the most central nodes based upon our different network measures.

Phenotype	***PheCode***	***Attribute***	***Value***
**Hypothyroidism NOS**	244.4	Degree	83
**Disorders of lipoid metabolism**	272	Degree	79
**Type 2 diabetes**	250.2	Degree	79
Diabetes mellitus	250	Degree	77
**Hyperlipidemia**	272.1	Degree	76

**Celiac disease**	557.1	Weighted Degree	$1.27*10^5$
Non-celiac intestinal malabsorption	557	Weighted Degree	$1.26*10^5$
**Hypothyroidism NOS**	244.4	Weighted Degree	$7.48*10^4$
**Hypothyroidism**	244	Weighted Degree	$7.39*10^4$
**Type 1 diabetes**	250.1	Weighted Degree	$6.53*10^4$
**Psoriasis**	696	Weighted Degree	$5.09*10^4$
Psoriasis NOS	696.4	Weighted Degree	$5.11*10^4$
Disorders of muscle, ligament, and fascia	728	Closeness Centrality	1.00
Fasciitis	728.7	Closeness Centrality	1.00
Other retinal disorders	362	Closeness Centrality	1.00
Skin cancer	172	Betweenness Centrality	$2.15*10^3$
**Disorders of lipoid metabolism**	272	Betweenness Centrality	$1.97*10^3$
**Hyperlipidemia**	272.1	Betweenness Centrality	$1.97*10^3$
Essential hypertension	401.1	Betweenness Centrality	$1.84*10^3$
Hypertension	401	Betweenness Centrality	$1.19*10^3$
Coronary atherosclerosis	411.4	Betweenness Centrality	$7.72*10^2$
Intestinal malabsorption	557	Eigenvector Centrality	1.00
**Celiac disease**	557.1	Eigenvector Centrality	1.00
**Hypothyroidism NOS**	244.4	Eigenvector Centrality	0.98
**Hypothyroidism**	244	Eigenvector Centrality	0.98
**Type 1 diabetes**	250.1	Eigenvector Centrality	0.95
**Type 2 diabetes**	250.2	Eigenvector Centrality	0.93
Rheumatoid arthritis	714.1	Eigenvector Centrality	0.89
Other inflammatory polyarthropathies	714	Eigenvector Centrality	0.89
**Psoriasis**	696	Eigenvector Centrality	0.86

Using the UKBB electronic health records, phi correlations[21] between pairs of phenotypes were calculated for 224 phenotypes in order to identify potential disease comorbidities. These associations were compared to the SNP-based edges in our DDN as a way of evaluating the extent to which genetic associations in PheWAS summary statistics match disease co-occurrences. Out of the 2189 edges for which phi correlations could be calculated, 1811 (82.73%) appeared in the DDN. This behavior suggests that our genetic associations identified by our PheWAS results serve as a reasonable approximation of disease co-occurrences.

The DDN we generated includes many disease connections identified in previous studies. In keeping with the DDN generated from the DiscovEHR biobank[7], our network identified connections among type 1 diabetes, rheumatoid arthritis, psoriasis, and multiple sclerosis. It also identified connections among hypothyroidism, type 2 diabetes, thyroid cancer, obesity, and rheumatoid arthritis. Furthermore, similar to the Disease Comorbidity Network[22] derived from hospitals across China, our DDN included edges between hypertension and hyperlipidemia, type 1 and type 2 diabetes, and diabetes mellitus. Finally, in keeping with a multimorbidity study performed on elderly patients in Tokyo[23], our DDN identified connections between hypertension, dyslipidemia, and coronary heart disease.

Finally, considering potential genetic associations between diseases, we find that our DDN displays relevant genetic associations between diseases, including rs544873's association with pulmonary heart disease, phlebitis and thrombophlebitis, hemorrhoids, circulatory disease, and diverticulosis[24], rs925488's association with thyroid cancer, nontoxic nodular and multinodular goiter, and hypothyroidism[24], and rs780094's association with diabetes and lipid metabolism.[25]

Testing

As a test of runtime for NETMAGE, we constructed DDNs from random subsets of the PheWAS data used to create the UKBB DDN and determined the time it took for each network to be generated. Five networks were each generated from collections of 50, 100, 250, 500, and 1000 phenotypes. These DDNs were constructed in both the Fruchterman-Reingold and Force Atlas 2 layouts from Gephi[13], resulting in a total of 50 graphs for runtime analysis. The average time to create a network seems to grow in $O(n^2)$ as the number of phenotypes increases (Table 3). This behavior makes sense, as the inclusion of additional nodes will tend to exponentially increase the number of edges assuming a low clustering coefficient in the network.

Table 3. A table of run times (in seconds) for DDN generation given input datasets with different numbers of phenotypes. These times measure how long it takes for the server to generate the network after the "submit" button has been clicked – in all instances, files have already been uploaded to the server. Upload speeds for files will vary depending on user bandwidth. Five replicates were performed for each count of phenotypes, and the mean and standard deviation of time is provided after each section.

Phenotype Count	**Server runtime (in seconds) to generate network after receiving HTTP request**													
	Fruchterman-Reingold Layout							***Force Atlas 2 Layout***						
	1	***2***	***3***	***4***	***5***	***Mean***	***SD***	***1***	***2***	***3***	***4***	***5***	***Mean***	***SD***
50	3.07	2.34	2.86	2.31	2.76	2.67	0.33	2.46	2.48	2.93	2.43	3.00	2.66	0.28
100	3.26	3.49	4.29	3.61	3.52	3.63	0.39	3.43	4.14	4.37	4.62	3.58	4.03	0.51
250	6.60	5.20	6.77	6.62	5.56	6.15	0.72	6.74	5.31	6.36	6.92	5.90	6.25	0.65
500	11.21	11.85	12.53	10.94	9.91	11.29	0.99	11.68	12.04	12.49	11.21	9.33	11.35	1.22
1000	28.27	28.77	30.19	27.01	29.52	28.75	1.22	29.37	28.35	29.84	27.23	30.23	29.00	1.22

Discussion and Conclusions

NETMAGE is a toolkit for the network-based interactive visualization of PheWAS summary data. The goal of this software is to improve the ease of visualization of genetic associations across diseases and to facilitate large-scale genetic analysis of the human diseasome.

Several future directions exist for NETMAGE. First is the inclusion of directionality in the network using beta values from PheWAS results. We will also allow for the selection of multiple nodes at once within the DDN – as of now, a user can select just one node to visualize its associations. The ability to select multiple nodes will allow clinicians to visualize genetic associations across triplets of disease. Furthermore, we hope to enhance NETMAGE to allow for the construction of gene-based DDNs by including SNP-to-gene mapping as a part of the website. We will also allow users the option to create SNP-SNP networks that depict edges between genetic variants based upon shared associations with phenotypes.

Ultimately**,** NETMAGE will give researchers and clinicians insight into the underlying genetic architecture of disease complications. The impact of our work will be a tool that allows for identification of new gene targets that can be investigated in follow-up studies of pleiotropy and drug discovery. We hope that this software will contribute to new potential discoveries in personalized medicine and that it helps facilitate the advancement of network medicine studies into the genetics of disease co-occurrences.

Availability of supporting source code and requirements

- Project name: NETMAGE
- Project home page: https://hdpm.biomedinfolab.com/netmage/
- Source code: https://github.com/dokyoonkimlab/netmage

- Operating system(s): Platform independent
- Programming language: Python, HTML, JavaScript
- Other requirements: None

Funding

This work has been supported by the NIGMS R01 GM138597.

Conflict of Interest: none declared.

Citations

1. Valderas JM, Starfield B, Sibbald B, Salisbury C, Roland M. Defining Comorbidity: Implications for Understanding Health and Health Services. *The Annals of Family Medicine*. 2009;7(4):357-363. doi:10.1370/afm.983

2. Bush WS, Oetjens MT, Crawford DC. Unravelling the human genome–phenome relationship using phenome-wide association studies. *Nat Rev Genet*. 2016;17(3):129-145. doi:10.1038/nrg.2015.36

3. Rubio-Perez C, Guney E, Aguilar D, et al. Genetic and functional characterization of disease associations explains comorbidity. *Sci Rep*. 2017;7(1):6207. doi:10.1038/s41598-017-04939-4

4. Denny J, Bastarache L, Roden D. Systematic comparison of phenome-wide association study of electronic medical record data and genome-wide association study data. *Nature Biotechnology*. 2013;31:1102-1111. doi:10.1038/nbt.2749

5. Barabási A-L, Gulbahce N, Loscalzo J. Network medicine: a network-based approach to human disease. *Nat Rev Genet*. 2011;12(1):56-68. doi:10.1038/nrg2918

6. Goh K-I, Cusick ME, Valle D, Childs B, Vidal M, Barabasi A-L. The human disease network. *Proceedings of the National Academy of Sciences*. 2007;104(21):8685-8690. doi:10.1073/pnas.0701361104

7. Verma A, Bang L, Miller J, et al. Human-Disease Phenotype Map Derived from PheWAS across 38,682 Individuals. *AJHG*. 2019;104(1):55-64. doi:10.1016/j.ajhg.2018.11.006

8. Gao XR, Huang H. PleioNet: a web-based visualization tool for exploring pleiotropy across complex traits. *Bioinformatics*. 2019;35(20):4179-4180. doi:10.1093/bioinformatics/btz179

9. Kortemeier E, Ramos P, Hunt K, Kim H, Hardiman G, Chung D. ShinyGPA: An interactive visualization toolkit for investigating pleiotropic architecture using GWAS datasets. *PLOS ONE*. 2018;13(1). doi:10.1371/journal.pone.0190949

10. George G, Gan S, Huang Y, et al. PheGWAS: a new dimension to visualize GWAS across multiple phenotypes. *Bioinformatics*. 2020;36(8):2500-2505. doi:10.1093/bioinformatics/btz944

11. Gagliano Taliun SA, VandeHaar P, Boughton AP, et al. Exploring and visualizing large-scale genetic associations by using PheWeb. *Nat Genet*. 2020;52(6):550-552. doi:10.1038/s41588-020-0622-5

12. Strayer N, Shirey-Rice J, Shyr Y, Denny J, Pulley J, Xu Y. PheWAS-ME: A web-app for interactive exploration of multimorbidity patterns in PheWAS. *medRXiv*. Published online June 2, 2020. doi:10.1101/19009480

13. Bastian M, Heymann S, Jacomy M. Gephi: An Open Source Software for Exploring and Manipulating Networks. In: Association for the Advancement of Artificial Intelligence; 2009.

14. Oxford Internet Institue. Interactive Visualizations. Published 2020. http://blogs.oii.ox.ac.uk/vis/

15. Jacomy A, Plique G. *Sigmajs*. http://sigmajs.org

16. Zhou W, Nielsen J, Fritsche L, et al. Efficiently controlling for case-control imbalance and sample relatedness in large-scale genetic association studies. *Nature Genetics*. 2018;50:1335-1341. doi:10.1038/s41588-018-0184-y

17. *UK Biobank*. https://www.ukbiobank.ac.uk

18. Wei W-Q, Bastarache LA, Carroll RJ, et al. Evaluating phecodes, clinical classification software, and ICD-9-CM codes for phenome-wide association studies in the electronic health record. Rzhetsky A, ed. *PLoS ONE*. 2017;12(7):e0175508. doi:10.1371/journal.pone.0175508

19. Altshuler D, Daly MJ, Lander ES. Genetic Mapping in Human Disease. *Science*. 2008;322(5903):881-888. doi:10.1126/science.1156409

20. Purcell S, Neale B, Todd-Brown K, et al. PLINK: A Tool Set for Whole-Genome Association and Population-Based Linkage Analyses. *The American Journal of Human Genetics*. 2007;81(3):559-575. doi:10.1086/519795

21. Hidalgo CA, Blumm N, Barabási A-L, Christakis NA. A Dynamic Network Approach for the Study of Human Phenotypes. Meyers LA, ed. *PLoS Comput Biol*. 2009;5(4):e1000353. doi:10.1371/journal.pcbi.1000353

22. Guo M, Yu Y, Wen T, et al. Analysis of disease comorbidity patterns in a large-scale China population. *BMC Med Genomics*. 2019;12(S12):177. doi:10.1186/s12920-019-0629-x

23. Mitsutake S, Ishizaki T, Teramoto C, Shimizu S, Ito H. Patterns of Co-Occurrence of Chronic Disease Among Older Adults in Tokyo, Japan. *Prev Chronic Dis*. 2019;16:180170. doi:10.5888/pcd16.180170

24. Zhou W, Brumpton B, Asvold B. GWAS of thyroid stimulating hormone highlights pleiotropic effects and inverse association with thyroid cancer. *Nature Communications*. 2020;11. doi:10.1038/s41467-020-17718-z

25. Bi M, Kao WH, Boerwinkle E, et al. Association of rs780094 in GCKR with Metabolic Traits and Incident Diabetes and Cardiovascular Disease: The ARIC Study. *PLOS ONE*. 2010;5. doi:10.1371/journal.pone.0011690
