# Supplementary material for: NETMAGE: A human disease phenotype map generator for the network-based visualization of phenome-wide association study results
Source: Gigascience. 2022 Feb 15;11:giac002. doi: 10.1093/gigascience/giac002 (PMC8848314; doi:10.1093/gigascience/giac002)
Supplement: giac002_GIGA-D-21-00220_Revision_1 [file giac002_giga-d-21-00220_revision_1.pdf]

GigaScience

NETMAGE: A Human Disease Phenotype Map Generator for the Network-based Visualization of PheWAS Results

--Manuscript Draft--

**Manuscript Number:**	GIGA-D-21-00220R1
**Full Title:**	NETMAGE: A Human Disease Phenotype Map Generator for the Network-based Visualization of PheWAS Results
**Article Type:**	Technical Note
**Funding Information:**	national institute of general medical sciences (R01 GM138597) — Dr. Dokyoon Kim
**Abstract:**	Background<br>Disease complications, the onset of secondary phenotypes given a primary condition, can exacerbate the long-term severity of outcomes. However, the exact cause of many of these cross-phenotype associations is still unknown. One potential reason is shared genetic etiology – common genetic drivers may lead to the onset of multiple phenotypes. A holistic, network-based view incorporating knowledge of other diseases and genetic associations will be required to uncover the exact basis of disease complications. Disease-disease networks (DDNs), where nodes represent diseases and edges represent associations between diseases, can provide an intuitive way of understanding the relationships between phenotypes. Using summary statistics from a phenome-wide association study (PheWAS), we can generate a corresponding DDN where edges represent shared genetic variants between diseases. Such a network can help us analyze genetic associations across the diseasome, the landscape of all human diseases, and identify potential genetic influences for disease complications.<br>Results<br>To improve the ease of network-based analysis of shared genetic components across phenotypes, we developed the humaN disEase phenoType MAp GEnerator (NETMAGE), a web-based tool that produces interactive DDN visualizations from PheWAS summary statistics. Users can search the map by various attributes and select nodes to view related phenotypes, associated variants, and various network statistics. As a test case, we used NETMAGE to construct a network from UK BioBank (UKBB) PheWAS summary statistic data. Our map correctly displayed previously identified disease comorbidities from the UKBB and identified concentrations of hub diseases in the endocrine/metabolic and circulatory disease categories. By examining the associations between phenotypes in our map, we can identify potential genetic explanations for the relationships between diseases and better understand the underlying architecture of the human diseasome. Our tool thus provides researchers with a means to identify prospective genetic targets for drug design, using network medicine to contribute to the exploration of personalized medicine.<br><br>Availability: Our service runs at https://hdpm.biomedinfolab.com. Source code can be downloaded from https://github.com/dokyoonkimlab/netmage.
**Corresponding Author:**	Dokyoon Kim<br>University of Pennsylvania<br>Philadelphia, PA UNITED STATES
**Corresponding Author Secondary Information:**	
**Corresponding Author's Institution:**	University of Pennsylvania
**Corresponding Author's Secondary Institution:**	
**First Author:**	Vivek Sriram
**First Author Secondary Information:**	
**Order of Authors:**	Vivek Sriram

Powered by Editorial Manager® and ProduXion Manager® from Aries Systems Corporation

	Manu Shivakumar
	Sang-Hyuk Jung
	Yonghyun Nam
	Lisa Bang
	Anurag Verma
	Seunggeun Lee
	Eun Kyung Choe
	Dokyoon Kim
**Order of Authors Secondary Information:**	

Response to Reviewers:

We appreciate the valuable comments of the anonymous reviewers which were helpful to improve the quality of the paper. The chances to our manuscript that reflect reviewers' comments are highlighted in 'red' in the following document.

1.Revision on Comments of Reviewer 1
2.Revision on Comments of Reviewer 2
3.Revision on Comments of Reviewer 3

[Revision on Comments of Reviewer 1]

[R1: Comment 1]
Generally well written and logical flow. Some minor errors (e.g. "an SNP" rather than "a SNP") and some headers could be improved for readability (e.g. "Testing" is vague; this section really only touches upon Run time).

[Response to Comment 1]
We correct the identified mistakes in our manuscript, fixing all instances of "an SNP" to "a SNP," as well as renaming the "Testing" section to "Runtime Analysis."

[R1: Comment 2]
Figure 1- Displaying a single Manhattan plot for "PheWAS Summary Statistics" is not very intuitive. It makes me think of a single GWAS rather than a phenome-wide set of GWAS run on a Biobank. Perhaps revise the image.

[Response to Comment 2]
Figure 1 has been revised to include a Manhattan plot corresponding to a PheWAS instead of a GWAS, with phenotypes instead of genes appearing along the x-axis of the plot.

[R1: Comment 3]
Is the disease-disease network only applicable to case/control studies? Could there be an extension to quantitative traits, and if so, would that be pertinent for discoveries?

[Response to Comment 3]
Disease-disease networks are indeed applicable to both binary and continuous phenotypes. In the case of quantitative traits, a PheWAS would be performed between genetic variants as independent variables and the continuous value of each phenotype as the outcome variable. Considering the associations between variants and such diseases may provide additional nuance into the strength of links between phenotypes. We have revised the Discussion and Conclusions section to include a mention of NETMAGE's applicability to quantitative traits.

[In the revised manuscript]
(6. Discussion and Conclusions)

…to facilitate large-scale genetic analysis of the human diseasome. While the UKBB data used for our case study consisted solely of binary phenotypes, NETMAGE can also be applied to quantitative traits. Indeed, in such a situation, the continuous value of the quantitative phenotype serves as the outcome variable in the PheWAS. This process provides a more detailed degree of association between the trait and genetic variants, suggesting a link between the variant and the severity of the phenotype as opposed to its presence or absence.

[R1: Comment 4]
The authors refer to "SNPs" throughout to define genetic variation. If the summary statistics contains another type of variation (e.g. indels), are those associations still used? If so, I would suggest using a more generic term to define the genetic variation.

[Response to Comment 4]
We thank the reviewer for identifying this over-simplification in our manuscript. Indeed, NETMAGE can use data involving any sort of genetic variation to generate a corresponding DDN. We replace the term "SNP" in our manuscript with "variant" or "genetic variant" as appropriate.

[In the revised manuscript]
(Abstract)
…Using summary statistics from a phenome-wide association study (PheWAS), we can generate a corresponding DDN where edges represent shared genetic variants between diseases…

…Users can search the map by various attributes and select nodes to view related phenotypes, associated variants, and various network statistics.

(1. Background)
…a phenome-wide association study (PheWAS) can be used to calculate a multitude of associations between phenotypes and genetic variants, such as single-nucleotide polymorphisms (SNPs) in an unbiased manner…

…In particular, a DDN that uses its edges to represent variants can be generated as a proxy to highlight potential shared genetic influences for diseases. Analyzing the topology of these genetics-based DDNs can provide insight into how inherited factors may drive the onset of disease complications…

(2. Purpose of the work)
…The network-based visualization of associations between variants and phenotypes can provide researchers…

… In particular, the resulting DDN is a projection of an undirected bipartite network of phenotypes and genetic variants, where nodes serve as diseases and edges serve as sets of common associated variants. Users can filter their input data by p-value and by minor allele frequency (MAF) to manipulate the rarity and significance of variants being used to generate the network. Furthermore, they can select nodes within the DDN to view information such as connected phenotypes, shared variants, and network statistics…

… users can follow up with phenotypic data in their corresponding EHRs to evaluate the predictive ability of genetics-based DDNs with respect to disease co-occurrences…

(Table 1)
…Allow users to search and create subsets of any produced networks by disease, by genetic variant, or by other network statistics.

(3. Implementation)
…Each row should correspond to a genetic variant, and the user can provide p-value…

…This file represents a dictionary of phenotype-to-variant mappings, where each phenotype serves as a key and each variant, p-value, MAF triplet serves as a value in a set…

Powered by Editorial Manager® and ProduXion Manager® from Aries Systems Corporation

…Based upon the p-value and MAF thresholds provided by the user, phenotype-variant mappings will be filtered to provide a final file containing a list of relevant variants for each disease…

… The weight of the edge is equal to the number of associated variants shared between the two phenotypes…

… Each row provides a distinct phenotype and a list of its associated variants…

… users can search the map for relevant phenotypes based upon any attributed defined, such as phenotype name, phenotype ID, variant name, node degree, and other parameters. In particular, the "search by variant" option allows users to find shared genetic variants between diseases.

…searchability of DDNs by both phenotype and genetic variant…

…providing a map of phenotypes to variants…

(Figure 2)
…Additionally, associated variants, connected phenotypes, and …

(6. Discussion and Conclusions)
…Furthermore, we hope to enhance NETMAGE to allow for the automated construction of gene-based DDNs from variant-based data by including variant-to-gene mapping as a part of the website. We will also allow users the option to create variant-variant networks that depict edges between genetic variants based upon shared associations with phenotypes …

[R1: Comment 5]
The discussion seems underdeveloped. Discussion of limitations rather than only future work would be helpful.

[Response to Comment 5]
We have revised the "Discussion" section to include a paragraph on current limitations of NETMAGE. The Discussion has also been extended to include a description of DDNs generated from continuous traits.

[In the revised manuscript]
(6. Discussion and Conclusions)
…The goal of this software is to improve the ease of visualization of genetic associations across diseases and to facilitate large-scale genetic analysis of the human diseasome. While the UKBB data used for our case study consisted of entirely binary phenotypes, NETMAGE is also applicable to quantitative traits. Indeed, in such a situation, the continuous value of the quantitative phenotype, such as a laboratory test measurement like A1C level, is used as the outcome variable in the PheWAS. This process provides a more detailed degree of association between the severity of the trait and genetic variants, as compared to the identification of associations between a presence or absence of the trait with variants. A key point to note regarding NETMAGE is that the output DDNs will provide only as much information as the input data. Indeed, NETMAGE is an exploratory tool intended to help visualize connections between diseases. Including summary PheWAS data that provides insight into the statistical associations between phenotypes will yield an associative map but will tell us nothing about causality. Associations identified through PheWAS are often spurious, so any sort of analyses performed on these data must take this information into consideration. Nevertheless, these kinds of associative visualizations are still useful for the study of disease and may help identify connections between phenotypes and genetic variants, generate new hypotheses, and suggest future experiments that can be conducted. For a visualization that gives stronger insight into the causal connections between traits, one could potentially input the results of a Mendelian Randomization experiment. Several future directions exist for NETMAGE. First is the inclusion of directionality in the network – as of now, DDNs produced by NETMAGE give no indication regarding the direction of association between phenotypes. Using beta values for the association

between phenotypes and genetic variants would be a useful inclusion, aiding in clinical interpretation of the network. We will also allow for the concurrent selection of multiple nodes within the DDN. The current NETMAGE user interface allows only one node to be selected at a time. The ability to select multiple nodes will allow clinicians to quickly identify if two phenotypes are associated in the network. We also hope to enhance NETMAGE to allow for the construction of gene-based DDNs from variant-based data by including variant-to-gene mapping as a part of the website. Finally, we will allow users to create variant-variant networks instead of disease-disease networks, which depict the connections between genetic variants (for instance, SNPs) based upon associations with phenotypes. Ultimately, NETMAGE will give researchers and clinicians insight into the underlying genetic architecture of disease complications…

[R1: Comment 6]
Case study-- The authors could improve the interpretability/discussion of the UKB PheWAS example. This is one of my largest concerns because the author state that the tool can help researchers and clinicians get insight into the underlying genetic architecture of disease complications; however, the case study part of the manuscript is quite technical and could be challenging to interpret for someone without network experience; e.g. Table 2.

[Response to Comment 6]
We very much appreciate the reviewer's comments regarding interpretability – we have edited Table 2 to simply list hub diseases identified through network centrality measures. Phenotypes identified according to multiple centrality measures are depicted in bold in the table. Furthermore, to aid with interpretability when exploring the DDN, we have included a new hyperlink in the "Information Pane" when a phenotype is selected. This link directs the user to a new window which depicts a histogram of diseases connected to the phenotype of interest, sorted in order of number of shared variants. This new feature should aid users in visualizing the significance of disease connections to a phenotype in the DDN, allowing for improved interpretability.

[In the revised manuscript]
(3. Implementation)
…Clicking on a node will highlight the node and all its first-degree neighbors. A variety of default attributes will be presented on the right side of the webpage as part of an "Information Pane." The user can also define other custom attributes, and these will be included in the Information Pane as well. If the user inputs data that include rsID-formatted SNPs, then NETMAGE will automatically hyperlink each SNP's ID to its corresponding dbSNP profile, allowing for further exploration of the variant's information. To aid with interpretation and visualization of disease associations, a hyperlink to a histogram of disease connections is also included in the Information Pane. For each phenotype, this histogram depicts first-degree disease neighbors sorted in order of the number of shared variants…

[R1: Comment 7]
Additionally, more details should be provided on the underlying summary statistics used (e.g. some details can be found on the About page of the HRC-imputed UKB PheWeb page: https://pheweb.org/UKB-SAIGE/about).

[Response to Comment 7]
We thank the reviewer for pointing us to this clearer description of our input dataset. We include additional details about our data in the "Case Study" section of the manuscript

[In the revised manuscript]
(4. Case Study)
…These data corresponded to 1,403 binary phenotypes expressed in terms of PheCodes. All 400,000 British individuals of European ancestry in the dataset were imputed using the Haplotype Reference Consortium panel, yielding 28 million imputed SNPs.11 SAIGE16, a generalized mixed model association test that uses the saddlepoint approximation to account for case-control imbalance, was used to generate summary statistics for each SNP, providing p-values of association between every SNP and every phenotype. This analysis was adjusted for genetic relatedness,

Powered by Editorial Manager® and ProduXion Manager® from Aries Systems Corporation

sex, birth year, and the first four principal components.11 All genomic positions are on GRCh37.11 Phenotypes that had a case count lower than 200 were dropped to keep more relevant diseases, yielding a total of 1075 traits for consideration…

[R1: Comment 8]
The authors list additional filtering that they performed on the summary statistics, but it appears that some details are missing. For instance, how many traits remain after the case count filtering is applied? Also, what is used as a reference for the LD-pruning in PLINK?

[Response to Comment 8]
We have revised the description of our filtration steps to include how many traits were included after case count filtering, as well as a mention of the reference panel used for LD-pruning.

[In the revised manuscript]
(4. Case Study)
…SAIGE was used to generate summary statistics for each variant, providing p-values of association between every variant and every phenotype. Phenotypes that had a case count lower than 200 were dropped to keep more relevant diseases, yielding a total of 1075 traits. Data were also filtered in order to select significant associated common variants, based upon the following thresholds: maximum p-value threshold of 5x10^-8, minimum MAF of 0.01, and LD-pruning through PLINK using the quality-controlled UKBB genetic data itself as our reference panel, with an R^2 of 0.2 and 250 kilobases for maximum search length. Removing nodes with degree 0 after the previously described filtration steps yielded a final network of 232 nodes and 2375 edges…

[R1: Comment 9]
Run time-- I am wondering why Table 3 (run time for subsets of the UKBB data) ends at 1000 phenotypes. It would be interesting to see the run time that is close to case example (e.g. possibly adding a column for the total number of phenotypes used in the UKBB DDN). Additionally, this section gives the impression that run time only depend on the number of phenotypes? I would assume that run time should also depend on the number of variants that were tested.

[Response to Comment 9]
We clarify in the text of our runtime section that increasing the number of variants under consideration will increase the runtime. We also include a new row in Table 3 that includes the runtime for the UKBB DDN case study for both the Fruchterman-Reingold and Force Atlas 2 layouts.

[In the revised manuscript]
(5. Runtime Analysis)
… This behavior makes sense, as runtime depends on not only the number of phenotypes included in the input data, but also the number of variants being tested. Indeed, assuming that each additional phenotype added to the network will include multiple associated variants, the inclusion of nodes will tend to exponentially increase the number of edges assuming a low clustering coefficient in the network…

[R1: Comment 10]
It is nice that on each page the authors have allowed users to download a pdf of the image and also the data behind the image (e.g. edge-map, node-map, etc.). The zoom-in feature for the visualization is also useful, as is the short video tutorial.

[Response to Comment 10]
We thank the reviewer for their comments regarding the software and website.

[R1: Comment 11]

Powered by Editorial Manager® and ProduXion Manager® from Aries Systems Corporation

	I think that the search bar would be more user-friendly if suggestions automatically came up when the user begins to type.<br>[Response to Comment 11]<br>Auto-completion for any sort of categorical variable (e.g. phenotype ID, associated SNP ID, and category) has been implemented in the NETMAGE tool. Now, as a user begins to type, NETMAGE will refer to all possible values in the input data and provide suggestions that the user can search.<br><br>[In the revised manuscript]<br>(3. Implementation)<br>…Users can search the map for relevant phenotypes based upon any attribute defined, such as phenotype name, phenotype ID, variant name, node degree, and other parameters. In particular, the "search by variant" option allows users to find shared genetic variants between diseases. The custom attributes provided by the user are also automatically incorporated into the search dropdown menu. Any categorical variables, such as disease name, disease category, or variant name, will include an auto-completion dropdown menu that dynamically updates as users type out their query terms…<br><br><br>[R1: Comment 12]<br>Additionally, displaying the list of "associated SNPs" in a (sortable and/or searchable) table (with some annotations, such as chr, position, closest gene, consequence, rather than just rsID) could be a neater and more informative way to show these data, rather than simply as it appears currently as a list in the "information pane".<br><br>[Response to Comment 12]<br>The inclusion of a dynamically updating table of SNP information for each phenotype is challenging to include in the current version of NETMAGE, particularly since users may not be uploading data purely corresponding to SNPs. Instead, we have revised the Information Pane's presentation of associated variant information to present SNP information in a more useful manner. If the user's input data includes variant IDs that are formatted in terms of rsIDs, then the variants will automatically be hyperlinked to their profiles on dbSNP. Otherwise, the list of associated variants stays as is. This behavior allows users to delve into the details of a SNP of interest. In the future, we will offer the ability to view a table of annotated SNP information for each phenotype based upon Annovar/VEP. We will also allow users to download a text file of associated variants for a phenotype of interest, including links to dbSNP if appropriate as well as Annovar annotations. We have raised a ticket on GitHub for this update, and it can be found at the following link: https://github.com/dokyoonkimlab/netmage/issues/20. For now, if users wish to download a list of variants associated with a phenotype, they can download the "Node Map" file to see the genetic associations for their desired disease.<br><br>[In the revised manuscript]<br>(3. Implementation)<br>…clicking on a node will highlight the node and all its first-degree neighbors. A variety of default attributes will be presented on the right side of the webpage as part of an "Information Pane." The user can also define other custom attributes, and these will be included in the Information Pane as well. If the user inputs data that include rsID-formatted SNPs, then NETMAGE will automatically hyperlink each SNP's ID to its corresponding dbSNP profile, allowing for further exploration of the variant's information. To aid with interpretation and visualization of disease associations, a hyperlink to a histogram of disease connections is also included in the Information Pane. For each phenotype, this histogram depicts first-degree disease neighbors sorted in order of the number of shared variants…<br><br>[R1: Comment 13]<br>My comment on interpretability for researchers and clinicians comes up again: I am not sure how useful/interpretable some of the search categories are for users to intuitively draw insights; for instance, number of triangles, page range, etc. I think the authors should really focus on the intuitiveness for the target audience so that the tool can have more impact.<br><br>[Response to Comment 13]

Powered by Editorial Manager® and ProduXion Manager® from Aries Systems Corporation

We appreciate the reviewer’s concern regarding interpretability in the presentation of the network information for the DDN. We prefer to keep all network statistics in the visualization, as it is unclear what piece of information might be most useful for users to consider. However, we include “information” icons next to each network statistic term that can be hovered over to provide a brief description of the utility of the variable. We hope that this inclusion helps clear up confusion surrounding network analysis in the DDN.

[Revision on Comments of Reviewer 2]

[R2: Comment 1]
I tried the web interface Human-Disease Phenotype Map (https://hdpm.biomedinfolab.com), which utilizes NETMAGE. I found that sometimes it takes some time for the network to appear. While the network is loaded, only the gray empty space with the side panel is shown. I recommend the authors to show the progress bar while loading the network, especially when it is first loaded, to avoid users to think that their web browser is frozen.

[Response to Comment 1]
The NETMAGE website has been updated to include a loading circle as the network is being generated.

[R2: Comment 2]
In the Search bar, it is not always trivial to guess what to enter, especially for Phenotype Name, Associated SNPs, and category. Auto-completion features for these variables will significantly facilitate users' convenience.

[Response to Comment 2]
Auto-completion for any sort of categorical variable (e.g. phenotype ID, associated SNP ID, and category) has been implemented in the NETMAGE tool. Now, as a user begins to type, NETMAGE will refer to all possible values in the input data and provide suggestions that the user can search.

[In the revised manuscript]
(3. Implementation)
…Users can search the map for relevant phenotypes based upon any attribute defined, such as phenotype name, phenotype ID, variant name, node degree, and other parameters. In particular, the “search by variant” option allows users to find shared genetic variants between diseases. The custom attributes provided by the user are also automatically incorporated into the search dropdown menu. Any categorical variables, such as disease name, disease category, or variant name, will include an auto-completion dropdown menu that dynamically updates as users type out their query terms…

[R2: Comment 3]
Meaning of edges is somewhat unclear to me. Are the existence and the weights of edges purely based on the number of shared SNPs or are they based on any statistical methods? When the weights of edges are calculated, are the marginal counts taken into account? The same number of shared SNPs can have different meanings when the disease to which this edge is connected has a small number of associated SNPs vs. a large number of associated SNPs. How is this factor considered?

[Response to Comment 3]
We very much appreciate this point noted by the reviewer. In the baseline version of the DDN, as described in Step 3 of the “Implementation” section, “The weight of the edge is equal to the number of associated variants shared between the two phenotypes.” However, as the reviewer mentions, the degree of the phenotypes in question can have a clear impact on the significance of an edge between two

Powered by Editorial Manager® and ProduXion Manager® from Aries Systems Corporation

diseases. To address this discrepancy, we have incorporated a “Marginalize edges” checkbox in the website. Users can specify if they want their edge weights to simply represent the number of shared variants between two diseases, or by selecting the checkbox, if they want the weight of the edge to be marginalized by the number of variants associated with each of the parent phenotypes.

[In the revised manuscript]
(3. Implementation)
…This file is used to generate an edge map and a node map. The edge map establishes all links in the network – each row corresponds to an edge from a source to a target. Depending on the user’s choice, the weight of the edge equals either the number of associated variants shared between the two phenotypes, or the marginalized fraction of variants (the number of variants that constitute the edge divided by the union of the individual sets of variants for both phenotypes). In addition, the node map represents a list of all nodes in the network…

[R2: Comment 4]
The network generated by the Human-Disease Phenotype Map (https://hdpm.biomedinfolab.com) is usually huge and complex with a large number of edges. As a result, it is often not straightforward to understand the generated network. This is partially relevant to the fact that the network layout is static, i.e., locations of nodes remain the same regardless of which subnetworks are chosen. If the network layout is optimized for each subnetwork, it should be much easier for users to understand the network architecture. Given this, I recommend the authors to consider updating the network layout interactively when a subnetwork is selected.

[Response to Comment 4]
We appreciate the reviewer’s suggestion to dynamically update the network layout when a subset of the network is chosen. This feature will require a considerable amount of work in terms of the structure of our code and will be handled in a future version of the software. We have raised a ticket on GitHub for this issue, and it can be found at this link: https://github.com/dokyoonkimlab/netmage/issues/19

[R2: Comment 5]
When a subnetwork is chosen, the "Information Pane" appears. In this pane, it might be helpful for users if the authors provide some quick help link for each network score, e.g., how to interpret PageRank scores, etc.

[Response to Comment 5]
We appreciate the reviewer’s suggestion for how to improve the interpretability of resulting networks. We have edited the NETMAGE Information Pane so that for each network statistic, an information icon can be hovered over that provides a brief description of the statistic’s purpose.

[R2: Comment 6]
In the "Information Pane", a long list of SNPs is provided for "Associated SNPs" but it is not easy to use this list. I recommend the authors to make it downloadable as a table so that users can do downstream analysis. In addition, it will significantly facilitate users' convenience if each SNP ID is chosen, it brings the user to the relevant database, e.g., dbSNP. In this way, users can easily check where it is located in the sense of chromosome, gene, exon/intron/promoter/intergenic, etc. Alternatively, the authors can consider to use a quick information table (SNP ID, gene name, exon/intron/promoter/intergenic) instead of simply providing as a list.

[Response to Comment 6]
We have revised the Information Pane’s presentation of associated variant information to present SNP information in a more useful manner. If the user’s input data includes variant IDs that are formatted in terms of rsIDs, then the variants will automatically be hyperlinked to their profiles on dbSNP. Otherwise, the list of associated variants stays as is. This behavior allows users to delve into the details of a SNP of interest. In the future, we will offer the ability to view a table of annotated SNP information for each

Powered by Editorial Manager® and ProduXion Manager® from Aries Systems Corporation

phenotype based upon Annovar/VEP. We will also allow users to download a text file of associated variants for a phenotype of interest, including links to dbSNP if appropriate as well as Annovar annotations. We have raised a ticket on GitHub for this update, and it can be found at the following link: https://github.com/dokyoonkimlab/netmage/issues/20. For now, if users wish to download a list of variants associated with a phenotype, they can download the “Node Map” file to see the genetic associations for their desired disease.

[In the revised manuscript]
(3. Implementation)
…clicking on a node will highlight the node and all its first-degree neighbors. A variety of default attributes will be presented on the right side of the webpage as part of an “Information Pane.” The user can also define other custom attributes, and these will be included in the Information Pane as well. If the user inputs data that include rsID-formatted SNPs, then NETMAGE will automatically hyperlink each SNP’s ID to its corresponding dbSNP profile, allowing for further exploration of the variant’s information. To aid with interpretation and visualization of disease associations, a hyperlink to a histogram of disease connections is also included in the Information Pane. For each phenotype, this histogram depicts first-degree disease neighbors sorted in order of the number of shared variants…

[Revision on Comments of Reviewer 3]
[R3: Comment 1]
A DDN based on true genetic associations is useful for understanding complex disease comorbidities and their shared genetic etiology (pleiotropy). An interactive web tool to explore such a complex networked information could be highly useful for the proposed purposes of this tool. However, the EHR/Biobank PheWAS associations data are statistical in nature and commonly with small effect sizes. The reported genetic associations often are not well understood at the mechanistic level, and many genetic associations are spurious. Although certain positive findings can be observed from the disease network generated by NETMAGE, it's of concern the general usability of the current implementation of the tool in order to facilitate novel applications in drug design and personalized medicine, which requires the genetic associations to best represent the underlying true causal mechanism. Further work is needed to verify the genetic associations reported from PheWAS to minimize the impact of spurious associations.

[Response to Comment 1]
We appreciate the reviewer’s comments regarding the implications of NETMAGE. The applicability of the data that go into the software will dictate the applicability of the resulting DDN. Indeed, with the results of a PheWAS, NETMAGE will be able to produce only an associative map of disease connections. We include a more thorough discussion of association vs. causation in our “Discussion and Conclusions” section.

[In the revised manuscript]
(6. Discussion and Conclusions)
…A key point to note regarding NETMAGE is that the output DDNs will provide only as much information as the input data. Indeed, NETMAGE is an exploratory tool intended to help visualize connections between diseases. Including summary PheWAS data that provides insight into the statistical associations between phenotypes will yield an associative map but will tell us nothing about causality. Associations identified through PheWAS are often spurious, so any sort of analyses performed on these data must take this information into consideration. Nevertheless, these kinds of associative visualizations are still useful for the study of disease and may help identify connections between phenotypes and genetic variants, as well as suggest future experiments that can be conducted. For a visualization that gives stronger insight into the causal connections between traits, one could potentially input the results of a Mendelian Randomization experiment…

[R3: Comment 2]
Network edges based on SNPs without considering the linkage disequilibrium (LD) between SNPs is misleading and could miss a significant portion of associations that should be linked between diseases if the LD correlations are considered. When construct the network using NETMAGE, the LD correlation between SNPs should be

Powered by Editorial Manager® and ProduXion Manager® from Aries Systems Corporation

considered.

[Response to Comment 2]
We thank the reviewer for identifying this gap in our software. While we had included a feature to account for an input LD file in our back-end software, we had failed to include it in the NETMAGE website itself. The option for LD pruning according to an input LD file is now incorporated into the NETMAGE website.

[In the revised manuscript]
(3. Implementation)
…Each row provides a distinct phenotype and a list of its associated variants. If input data have not already been pruned for linkage disequilibrium (LD), then users can provide an LD-mapping file that gives mappings between each variant to blocks of LD. NETMAGE will then clump SNPs according to their specified LD blocks, ensuring that associations that should be linking phenotypes together are present in the map. Users can also provide an input disease category mapping file so that each row of the node map now represents the disease and its category…

[R3: Comment 3]
For the reported DDN and its statistics to be relevant to true disease - disease relationships, the quality of disease diagnosis using Phecode should be considered. Phecodes are based on ICD codes that are known to be noisy. The accuracy of ICD can be as low as only 50%. Ignoring this limitation and treating disease diagnoses from Phecodes as gold standards or as precise and accurate may result in irrelevant and misleading findings.

[Response to Comment 3]
This point regarding the appropriateness of Phecodes is extremely relevant. We include a small description of the limitations of Phecodes in our "Case Study" section.

[In the revised manuscript]
(4. Case Study)
…and rs780094's association with diabetes and lipid metabolism. One potential issue in terms of the conclusions that can be drawn from our UKBB DDN is the use of "PheCodes" as a method of defining phenotypes. PheCodes are defined according to ICD codes, but the accuracy of these codes for disease diagnosis is known to be questionable. Given such inaccuracies, users must be wary when treating PheCode or ICD-based diagnoses as a gold standard, as doing so may lead to inaccurate conclusions…

[R3: Comment 4]
Phecodes are hierarchical. For example, parent codes are three digits (008), and each additional digit after decimal point indicates a subset of ICD codes of the parent code (008.5 and 008.52). So here a code 008.52 implies 008.5 also 008. What's the impact of this hierarchy to the NETMAGE network and the inferences to be made based on the network?

[Response to Comment 4]
We agree with the reviewer that hierarchy between phenotypes may influence resulting DDNs. In our case study, the data we make use of includes mostly upper hierarchy phenotypes. More detailed hierarchical phenotypes are absent from the data. Users should be careful to avoid extensive hierarchical structure in their input data when generating DDNs through NETMAGE. We include a description of this facet in the "Case Study" section of our manuscript.

[In the revised manuscript]
(4. Case Study)
…Another aspect of the use of PheCodes for phenotype definitions is their hierarchical nature. Digits that appear after decimal points correspond to subsets of phenotypes compared to the parent code that appears before the decimal. In our case study, the data we make use of include mostly upper hierarchy phenotypes. More detailed hierarchical phenotypes are for the most part absent from our network. Users should be careful about including extensive hierarchical structure in their input data when

Powered by Editorial Manager® and ProduXion Manager® from Aries Systems Corporation

generating DDNs through NETMAGE. Including phenotypes that are essentially identical to one another will introduce unnecessary nodes and edges in the network, in the process clouding more significant disease connections…

[R3: Comment 5]
On Page 9, you said "Out of the 2189 edges for which phi correlations could be calculated, 1811 (82.73%) appeared in the DDN. This behavior suggests that our genetic associations identified by our PheWAS results serve as a reasonable approximation of disease co-occurrences". This is expected because both phi correlation and PheWAS analyses were performed on the same dataset. If a pair of disease highly co-occur in the dataset, you would expect a strong correlation on their genetic associations analyzed on the same dataset. However, it may not be generalizable that the genetic associations from PheWAS are a reasonable approximation to disease co-occurrences.

[Response to Comment 5]
We thank the reviewer for pointing out this flaw in our analysis of the DDN. We remove this analysis, and instead include a paragraph in our “Case Study” section that describes comparison to external EHR comorbidities as an area of future work.

[In the revised manuscript]
(4. Case Study)
…and rs780094’s association with diabetes and lipid metabolism. In terms of future work for this case study, it would be interesting to compare the edges in our DDN with known disease comorbidities. We can take disease occurrence data from an external electronic health record and evaluate phi correlations between all pairs of phenotypes. Comparison of these co-occurrences to the genetic associations in our PheWAS may give us an indication if the DDN is a reasonable representation of disease connections…

[R3: Comment 6]
The disease-SNP relationships from the PheWAS analysis result are bipartite. Even though NETMAGE focuses on the projected disease-disease network, the information about how specific SNPs link to their corresponding disease pairs is important. For example, in your UKBB-based network (https://hdpm.biomedinfolab.com/ddn/ukbb), when a specific disease is selected, a subgraph of the selected disease and other disease linked to the selected one are showing, but only a lump of SNPs without linking to their specific disease pair is provided. This is not helpful.

[Response to Comment 6]
We appreciate this comment from the reviewer, and we agree that selecting a single disease in our DDN does not provide insight into the links between variants and their corresponding disease pairs. For this purpose, we recommend that the user makes use of the “Search by SNP” feature to identify in which disease pairs the variant is involved. As a future extension of NETMAGE, we will offer the ability to generate variant-variant or gene-gene networks, which will make it easier to visualize how variants connect to diseases.

[R3: Comment 7]
Also annotating those SNPs their genetic context could be very useful for users to quickly grasp the nature of the genetic associations in the subgraph.

[Response to Comment 7]
We have revised the Information Pane’s presentation of associated variant information to present SNP information in a more useful manner. If the user’s input data includes variant IDs that are formatted in terms of rsIDs, then the variants will automatically be hyperlinked to their profiles on dbSNP. Otherwise, the list of associated variants stays as is. This behavior allows users to delve into the details of a SNP of interest. In the future, we will offer the ability to view a table of annotated SNP information for each phenotype based upon Annovar/VEP. We will also allow users to download a text file

Powered by Editorial Manager® and ProduXion Manager® from Aries Systems Corporation

	of associated variants for a phenotype of interest, including links to dbSNP if appropriate as well as Annovar annotations. We have raised a ticket on GitHub for this update, and it can be found at the following link: https://github.com/dokyoonkimlab/netmage/issues/20. For now, if users wish to download a list of variants associated with a phenotype, they can download the “Node Map” file to see the genetic associations for their desired disease.<br><br>[In the revised manuscript]<br>(3. Implementation)<br>…clicking on a node will highlight the node and all its first-degree neighbors. A variety of default attributes will be presented on the right side of the webpage as part of an “Information Pane.” The user can also define other custom attributes, and these will be included in the Information Pane as well. If the user inputs data that include rsID-formatted SNPs, then NETMAGE will automatically hyperlink each SNP’s ID to its corresponding dbSNP profile, allowing for further exploration of the variant’s information. To aid with interpretation and visualization of disease associations, a hyperlink to a histogram of disease connections is also included in the Information Pane. For each phenotype, this histogram depicts first-degree disease neighbors sorted in order of the number of shared variants…
**Additional Information:**	
**Question**	**Response**
Are you submitting this manuscript to a special series or article collection?	No
**Experimental design and statistics**<br><br>Full details of the experimental design and statistical methods used should be given in the Methods section, as detailed in our Minimum Standards Reporting Checklist. Information essential to interpreting the data presented should be made available in the figure legends.<br><br>Have you included all the information requested in your manuscript?	Yes
**Resources**<br><br>A description of all resources used, including antibodies, cell lines, animals and software tools, with enough information to allow them to be uniquely identified, should be included in the Methods section. Authors are strongly encouraged to cite Research Resource Identifiers (RRIDs) for antibodies, model organisms and tools, where possible.<br><br>Have you included the information requested as detailed in our Minimum	Yes

Standards Reporting Checklist?	
**Availability of data and materials**<br><br>All datasets and code on which the conclusions of the paper rely must be either included in your submission or deposited in publicly available repositories (where available and ethically appropriate), referencing such data using a unique identifier in the references and in the “Availability of Data and Materials” section of your manuscript.<br><br>Have you have met the above requirement as detailed in our Minimum Standards Reporting Checklist?	Yes

Powered by Editorial Manager® and ProduXion Manager® from Aries Systems Corporation

Manuscript

Click here to access/download;Manuscript;NETMAGE_revisions_gigaScience

NETMAGE: A Human Disease Phenotype Map Generator for the Network-based Visualization of PheWAS Results

[1] Department of Biostatistics, Epidemiology & Informatics, Perelman School of Medicine, University of Pennsylvania, Philadelphia, PA 19104, USA

[2] Department of Digital Health, SAIHST, Sungkyunkwan University, Samsung Medical Center, Seoul 06355, Republic of Korea

[3] Ultragenyx Pharmaceutical, Novato, CA 94949, USA

[4] Department of Medicine, Division of Translational Medicine and Human Genetics, Perelman School of Medicine, University of Pennsylvania, Philadelphia, PA 19104, USA

[5] Graduate School of Data Science, Seoul National University, Seoul 08826, Republic of Korea

[6] Institute for Biomedical Informatics, University of Pennsylvania, Philadelphia, PA 19104, USA

Corresponding Author: Dokyoon Kim Email: dokyoon.kim@pennmedicine.upenn.edu

Dokyoon Kim [0000-0002-4592-9564];
Vivek Sriram [0000-0003-3759-2911];
Manu Shivakumar [0000-0003-4733-7375];
Sang-Hyuk Jung [0000-0003-4116-3327];
Yonghyun Nam [0000-0003-2963-2151];
Lisa Bang [0000-0003-2502-858X];
Anurag Verma [0000-0002-5063-9107];
Seunggeun Lee [0000-0002-8097-3878];
Eun Kyung Choe [0000-0002-7222-1772];

Abstract

Background

Disease complications, the onset of secondary phenotypes given a primary condition, can exacerbate the long-term severity of outcomes. However, the exact cause of many of these cross-phenotype associations is still unknown. One potential reason is shared genetic etiology – common genetic drivers may lead to the onset of multiple phenotypes. A holistic, network-based view incorporating knowledge of other diseases and genetic associations will be required to uncover the exact basis of disease complications. Disease-disease networks (DDNs), where nodes represent diseases and edges represent associations between diseases, can provide an intuitive way of understanding the relationships between phenotypes. Using summary statistics from a phenome-wide association study (PheWAS), we can generate a corresponding DDN where edges represent shared genetic variants between diseases. Such a network can help us analyze genetic associations across the diseasome, the landscape of all human diseases, and identify potential genetic influences for disease complications.

Results

To improve the ease of network-based analysis of shared genetic components across phenotypes, we developed the humaN disEase phenoType MAp GEnerator (NETMAGE), a web-based tool that produces interactive DDN visualizations from PheWAS summary statistics. Users can search the map by various attributes and select nodes to view related phenotypes, associated variants, and various network statistics. As a test case, we used NETMAGE to construct a network from UK BioBank (UKBB) PheWAS summary statistic data. Our map correctly displayed previously identified disease comorbidities from the UKBB and identified concentrations of hub diseases in the endocrine/metabolic and circulatory disease categories. By examining the associations between phenotypes in our map, we can identify potential genetic explanations for the relationships between diseases and better understand the underlying architecture of the human diseasome. Our tool thus provides researchers with a means to identify prospective genetic targets for drug design, using network medicine to contribute to the exploration of personalized medicine. NETMAGE is available to use at https://hdpm.biomedinfolab.com. Source code can be downloaded from https://github.com/dokyoonkimlab/netmage.

Keywords

Disease-disease network; PheWAS; comorbidity; disease complication; network medicine

1. Background

Disease complications refer to the onset of secondary phenotypes given a primary condition, while disease comorbidities refer to the co-occurrent presence or onset of multiple diseases [1]. Both forms of disease association can exacerbate the long-term severity of disease, and they vary drastically from phenotype to phenotype [1]. However, their causes are still not well understood. One potential reason for these cross-phenotype associations [2] could be shared genetic etiology – the same genetic drivers may cause multiple symptoms to appear over time [3].

Electronic health record (EHR)-linked biobanks capture both clinical and genetic information for large populations of patients [4]. These repositories contain both genetic and longitudinal phenotype data, including DNA samples, disease histories, laboratory measurements, lifestyle habits, and demographic information [4]. Given an EHR-linked biobank as input, a phenome-wide association study (PheWAS) can be used to calculate a multitude of associations between phenotypes and genetic variants, such as single-nucleotide polymorphisms (SNPs), in an unbiased manner [4].

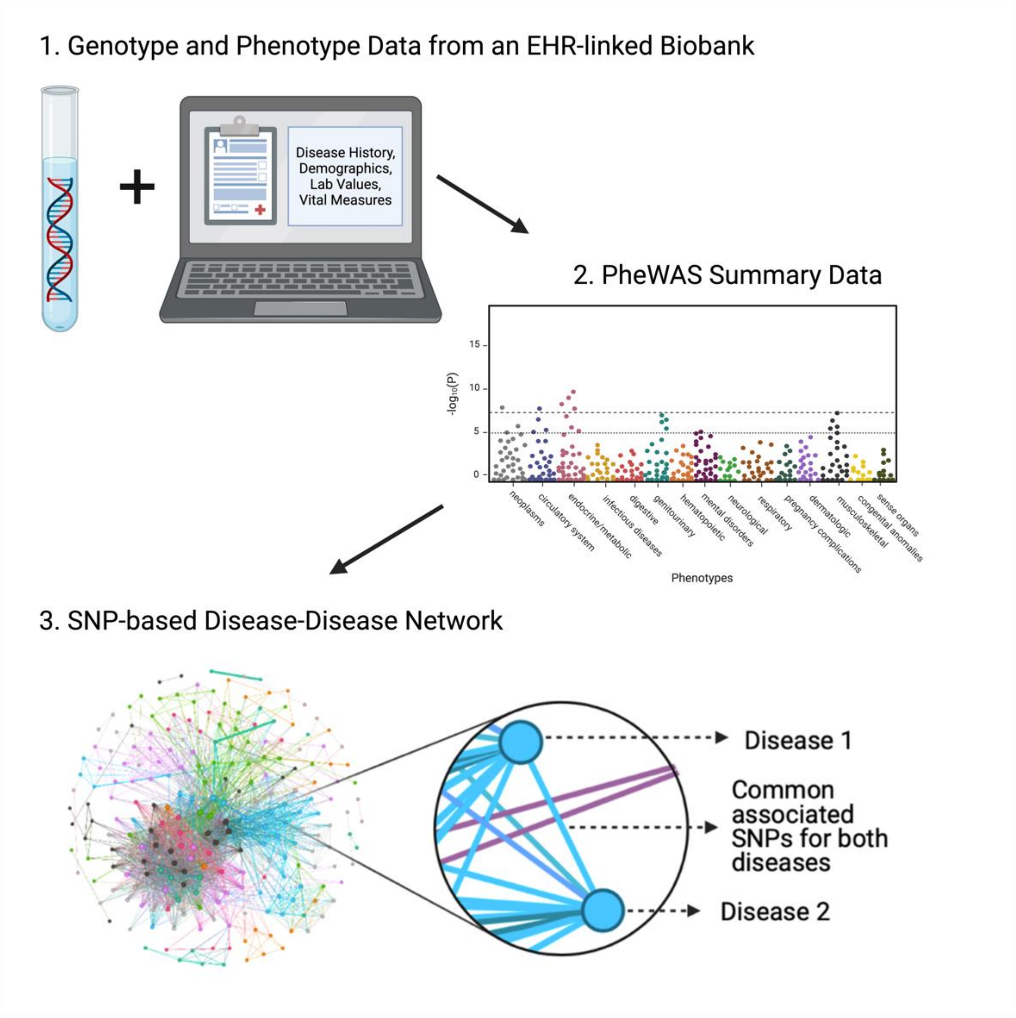

Figure 1. A depiction of the process for creating a SNP-based DDN. A PheWAS can be run on data from an EHR-linked biobank to calculate p-values of associations between a variety of single-nucleotide polymorphisms (SNPs) and phenotypes. The summary statistics from this PheWAS lend themselves to a DDN, where nodes represent diseases and edges represent common associated SNPs between diseases. Figure created with BioRender.com.

A holistic network-based view involving disorders across the diseasome will be required to translate these genetic correlations into an understanding of disease co-occurrences [5]. Disease-disease networks (DDNs), where nodes represent diseases and edges represent connections between diseases, can provide an intuitive way to understand the relationships between phenotypes [6, 7]. In particular, a DDN that uses its edges to represent variants can be generated as a proxy to highlight potential shared genetic influences for diseases. Analyzing the topology of these genetics-based DDNs can provide insight into how inherited factors may drive the onset of disease complications.

2. Purpose of the work

The network-based visualization of associations between variants and phenotypes can provide researchers and clinicians with a potential way to understand the genetic basis of disease interactions. In particular, the growth of available EHR-linked biobanks across institutions presents a trove of data that have yet to be mined from a "network medicine" perspective [5]. A variety of tools currently exist to depict PheWAS statistics, including PleioNet [8], ShinyGPA [9], PheGWAS [10], PheWeb [11], and PheWAS-ME [12] (Table 1). However, to the best of our knowledge, none of these packages allows for the creation of interactive, searchable DDNs from user-provided PheWAS summary data.

Table 1. A comparison of NETMAGE to other toolkits that currently exist for the visualization of PheWAS summary statistics.

Software Name	***Allows users to upload desired PheWAS results for analysis***	***Allows for interactive investigation of cross-phenotype associations***	***Generates a network visualization of genetic associations between phenotypes***	***Allows users to search and create subsets of any produced networks by disease, by genetic variant, or by other network statistics***
**PleioNet**		x	x	x
**ShinyGPA**	x	x		x
**PheGWAS**	x	x		N/A
**PheWAS-Me**	x	x		x
**PheWeb**	x	x		N/A

**NETMAGE**	x	x	x	x

The humaN disEase phenoType MAp GEnerator (NETMAGE) addresses this need. NETMAGE (NETMAGE, RRID:SCR_021843) is a web-based tool that allows users to upload any PheWAS summary statistics and generate corresponding interactive networks. In particular, the resulting DDN is a projection of an undirected bipartite network of phenotypes and genetic variants, where nodes serve as diseases and edges serve as sets of common associated variants [6]. Users can filter their input data by p-value and by minor allele frequency (MAF) to manipulate the rarity and significance of variants being used to generate the network. Furthermore, they can select nodes within the DDN to view information such as connected phenotypes, shared variants, and network statistics (Figure 2).

NETMAGE will serve as a step toward mass network-based analysis of PheWAS data. The interactive, graph-based representation of these summary statistics will help researchers visualize comorbidities as well as identify genetic variants that may potentially lead to the onset of disease complications. Furthermore, because NETMAGE facilitates the analysis of PheWAS data from individual EHR-linked biobanks, users can follow up with phenotypic data in their corresponding EHRs to evaluate the predictive ability of genetics-based DDNs with respect to disease co-occurrences. NETMAGE will allow us to gain a deeper understanding of the underlying genetic architecture of disease interaction.

3. Implementation

We used Gephi (Gephi, RRID:SCR_004293) [13], an open-source network visualization software package, as well as InteractiveVis [14], a framework built over sigma.js [15] for the interactive visualization of geospatial data, as a base for the implementation of NETMAGE. These packages were extended to create a web interface for the generation of network visualizations. We implemented a web server backend to accept the files uploaded by the user and then parse and generate the network using the Gephi toolkit. We deployed the server on Amazon Web Service (AWS) infrastructure, and it is available for use at the website [16]. We also enhanced the software to automatically parse all attributes provided in the input data and turn them into options for filtration and search. The NETMAGE pipeline works as follows:

1. **Users upload their PheWAS summary statistic files to our website**. Each row should correspond to a genetic variant, and the user can provide p-value and MAF information if they want to filter their data using NETMAGE. The data can be uploaded either as a single

file where the phenotype name is included in each row or separate files where each file corresponds to a distinct phenotype.

2. **NETMAGE converts PheWAS summary data into an intermediate *disease_snpmap.netmage* file.** This file represents a dictionary of phenotype-to-variant mappings, where each phenotype serves as a key and each variant, p-value, MAF triplet serves as a value in a set. To create a DDN from the same data in the future, the user can simply upload the *disease_snpmap.netmage* file instead of re-uploading the original PheWAS data by using the "Upload netmage file" option.
3. **The *disease_snpmap.netmage* file is converted into a corresponding node and edge map.** Based upon the p-value and MAF thresholds provided by the user, phenotype-variant mappings will be filtered to provide a final file containing a list of relevant variants for each disease. This file is used to generate an edge map and a node map. The edge map establishes all links in the network – each row corresponds to an edge from a source to a target. Depending on the user's choice, the weight of the edge equals either the number of associated variants shared between the two phenotypes, or the marginalized fraction of variants (the number of variants that constitute the edge divided by the union of the individual sets of variants for both phenotypes). In addition, the node map represents a list of all nodes in the network. Each row provides a distinct phenotype and a list of its associated variants. If input data have not already been pruned for linkage disequilibrium (LD), then users can provide an LD-mapping file that gives mappings between each variant to blocks of LD. NETMAGE will then clump SNPs according to their specified LD blocks, ensuring that associations that should be linking phenotypes together are present in the map. Users can also provide an input disease category mapping file so that each row of the node map now represents the disease and its category.
4. **The node and edge maps are used to create a two-dimensional mapping of the network.** Through the Gephi and InteractiveVis frameworks, each disease is mapped to a two-dimensional space to visualize the DDN. Within the NETMAGE webpage, users can specify parameters including network layout, node size, and edge thickness to edit the aesthetics of the resulting graph.

Given a resulting network, NETMAGE offers the following features:

- **Node Selection**: clicking on a node will highlight the node and all its first-degree neighbors. A variety of default attributes will be presented on the right side of the webpage as part of an "Information Pane." The user can also define other custom attributes, and

these will be included in the Information Pane as well. If the user inputs data that include rsID-formatted SNPs, then NETMAGE will automatically hyperlink each SNP's ID to its corresponding dbSNP profile [17], allowing for further exploration of the variant's information. To aid with interpretation and visualization of disease associations, a hyperlink to a histogram of disease connections is also included in the Information Pane. For each phenotype, this histogram depicts first-degree disease neighbors sorted in order of the number of shared variants.

- **Search**: users can search the map for relevant phenotypes based upon any attribute defined, such as phenotype name, phenotype ID, variant name, node degree, and other parameters. In particular, the "search by variant" option allows users to find shared genetic variants between diseases. The custom attributes provided by the user are also automatically incorporated into the search dropdown menu. Any categorical variables, such as disease name, disease category, or variant name, will include an auto-completion dropdown menu that dynamically updates as users type out their query terms.
- **Highlighting**: groups of nodes within the same disease category can be highlighted to visualize associations within groups. These categories are established according to the user-provided input disease category file.

Key strengths of NETMAGE include the automated creation of DDNs from user input for the visualization of a multitude of datasets, searchability of DDNs by both phenotype and genetic variant, and interactivity with the nodes of the DDN. These aspects allow users to focus on specific genetic associations by visualizing subsets of the map. Generated networks can be interacted with online or downloaded in a static format. NETMAGE allows users to download an image of the network as a PDF file or download the data corresponding to the network, including the intermediate *disease_snpmap.netmage* file (providing a map of phenotypes to variants, including p-value and MAF information if given by the user), node and edge map files (providing all nodes in the network along with their attributes, as well as all edges in the network respectively), and a final *data.json* file (providing the two-dimensional mapping of the elements in network). The node and edge map files, as well as the *data.json* file, can all be visualized and edited locally within Gephi. The *data.json* file can also be directly hosted by users on any web server.

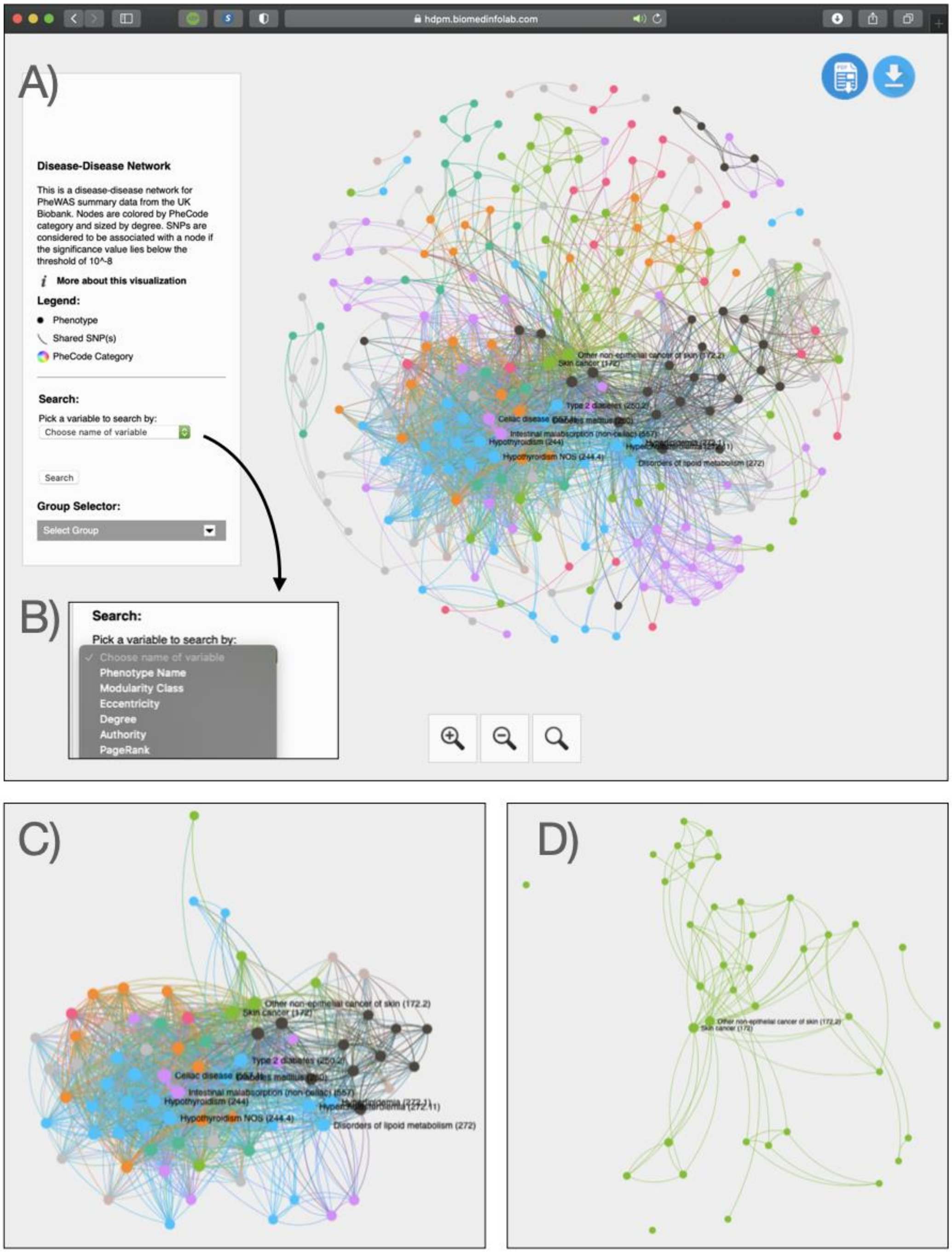

Figure 2. A depiction of the NETMAGE visualization tool. (A) The sidebar of the visualization gives a description of the map. It also includes a search dropdown and a group selector dropdown menu. (B) Variables are automatically read from the input data and included as options for search. (C) Clicking on a node reduces the displayed map to only the chosen node and its direct connections. Additionally, associated variants, connected phenotypes, and network statistics are presented to the right of the window when a node is selected. This graph corresponds to the subnetwork for type 2 diabetes (D) All nodes within

a single disease category can be visualized at once using the Group Selector. Here, we display all neoplasm phenotypes.

4. Case Study

As a demonstration of the abilities of NETMAGE, we applied our software to SAIGE [18] -analyzed UK Biobank [19] (UKBB) PheWAS data. The current version of the DDN is hosted at the website [20]. These data corresponded to 1,403 binary phenotypes expressed in terms of PheCodes [21]. All 400,000 British individuals of European ancestry in the dataset were imputed using the Haplotype Reference Consortium panel, yielding 28 million imputed SNPs [11]. SAIGE [18], a generalized mixed model association test that uses the saddlepoint approximation to account for case-control imbalance, was used to generate summary statistics for each SNP, providing p-values of association between every SNP and every phenotype. This analysis was adjusted for genetic relatedness, sex, birth year, and the first four principal components[11]. All genomic positions are on GRCh37 [11]. Phenotypes that had a case count lower than 200 were dropped to keep more relevant diseases, yielding a total of 1075 traits for consideration. Data were also filtered in order to select significantly associated common variants, based upon the following thresholds: maximum p-value threshold [22] of $5x10^{-8}$, minimum MAF of 0.01, and LD-pruning through PLINK [23] length using the quality-controlled UKBB genetic data itself as our reference panel, with an R^2 of 0.2 and 250 kilobases for maximum search.

Removing nodes with degree 0 after the previously described filtration steps yielded a final network of 232 nodes and 2375 edges. Degrees of nodes ranged from 1 to 84. The average degree was 20.47 and the average weighted degree was 1657.17. 68% (158/232) nodes had lower degrees than the average degree, implying a scale-free nature of the network (Figure 3) [5]. Furthermore, the diameter of the network was 7 while the average path length was 2.70, suggesting the small-world property for the network [5]. 570 edges (24%) connect diseases of the same category while 1,805 edges (76%) connect diseases of different categories, indicating that the genetic associations we identified appeared mostly across disease classes. Modularity analysis yielded 18 different clusters, ranging from size 2 to 72. There was also extensive variation in terms of the disease categories present for each module, again suggesting that genetic associations with phenotypes are not specific to disease class. Finally, the average clustering coefficient was 0.782, meaning that the network lacks extensive local clustering [5].

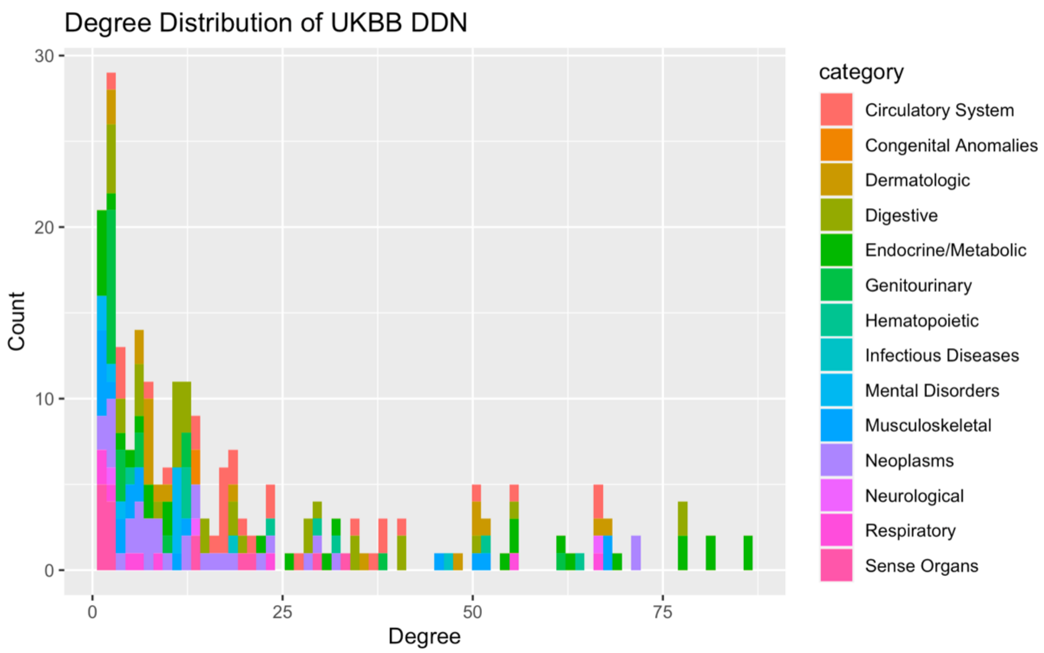

Figure 3. A histogram of degree distributions for the UKBB DDN. This distribution follows the power law, suggesting a scale-free property for the network. We also see that disease categories fail to follow specific trends based upon the degree of the disease.

Degree, weighted degree, closeness centrality, betweenness centrality, and eigenvector centrality were all used to identify hub diseases in the DDN 5]. Diseases with the highest degree included hyperlipidemia (272.1), disorders of lipoid metabolism (272), type 2 diabetes (250.2), diabetes mellitus (250), and hypothyroidism (244.4). Diseases with the highest weighted degree included celiac disease (557.1), non-celiac intestinal malabsorption (557), hypothyroidism (244), type 1 diabetes (250.1), and psoriasis (696 and 696.4). Highest closeness centrality phenotypes included disorders of muscle, ligament, and fascia (728), fasciitis (728.7), and other retinal disorders (362), and highest betweenness centrality phenotypes included disorders of lipoid metabolism (272), hyperlipidemia (272.1), skin cancer (172), coronary atherosclerosis (411.4), hypertension (401), and essential hypertension (401.1). Finally, highest eigenvector centrality diseases included intestinal malabsorption and celiac disease (557 and 557.1), hypothyroidism (244.4 and 244), type 2 diabetes (250.2), type 1 diabetes (250.1), psoriasis (696), and rheumatoid arthritis and other inflammatory polyarthropathies (714.1 and 714). Based upon these results, it appears that endocrine/metabolic and circulatory diseases seem to have the most influence in our DDN (Table 2).

Table 2. A table of hub phenotypes in the UKBB DDN. Centrality measures used to identify these phenotypes included degree, weighted degree, closeness centrality, betweenness centrality, and eigenvector centrality. Diseases marked in bold appear multiple times as the most central nodes based upon our different network measures. Refer to Table S1 in the Supplementary Data to see the exact centrality measures that identified each phenotype to be a hub.

Phenotype	***PheCode***	***Disease Category***
Skin cancer	172	Neoplasm
Diabetes mellitus	250	Endocrine/Metabolic
**Hypothyroidism**	244	Endocrine/Metabolic
**Hypothyroidism NOS**	244.4	Endocrine/Metabolic
**Type 1 diabetes**	250.1	Endocrine/Metabolic
**Type 2 diabetes**	250.2	Endocrine/Metabolic
**Disorders of lipoid metabolism**	272	Endocrine/Metabolic
**Hyperlipidemia**	272.1	Endocrine/Metabolic
Other retinal disorders	362	Sense Organs
Hypertension	401	Circulatory System
Essential hypertension	401.1	Circulatory System
Coronary atherosclerosis	411.4	Circulatory System
Non-celiac intestinal malabsorption	557	Digestive
**Celiac disease**	557.1	Digestive
**Psoriasis**	696	Dermatologic
Psoriasis NOS	696.4	Dermatologic
Other inflammatory polyarthropathies	714	Musculoskeletal
Rheumatoid arthritis	714.1	Musculoskeletal
Disorders of muscle, ligament, and fascia	728	Musculoskeletal
Fasciitis	728.7	Musculoskeletal

The DDN we generated includes many disease connections identified in previous studies. In keeping with the DDN generated from the DiscovEHR biobank [7], our network identified connections among type 1 diabetes, rheumatoid arthritis, psoriasis, and multiple sclerosis. It also identified connections among hypothyroidism, type 2 diabetes, thyroid cancer, obesity, and rheumatoid arthritis. Furthermore, similar to the Disease Comorbidity Network [24] derived from

hospitals across China, our DDN included edges between hypertension and hyperlipidemia, type 1 and type 2 diabetes, and diabetes mellitus. Finally, in keeping with a multimorbidity study performed on elderly patients in Tokyo [25], our DDN identified connections between hypertension, dyslipidemia, and coronary heart disease.

Finally, considering potential genetic associations between diseases, we find that our DDN displays relevant genetic associations between diseases, including rs544873's association with pulmonary heart disease, phlebitis and thrombophlebitis, hemorrhoids, circulatory disease, and diverticulosis [26], rs925488's association with thyroid cancer, nontoxic nodular and multinodular goiter, and hypothyroidism [24], and rs780094's association with diabetes and lipid metabolism [27].

One potential issue in terms of the conclusions that can be drawn from our UKBB DDN is the use of "PheCodes" as a method of defining phenotypes. PheCodes are defined according to ICD codes, but the accuracy of these codes for disease diagnosis is known to be questionable. Given such inaccuracies, users must be wary when treating PheCode or ICD-based diagnoses as a gold standard, as doing so may lead to inaccurate conclusions. Another aspect of the use of PheCodes for phenotype definitions is their hierarchical nature. Digits that appear after decimal points correspond to subsets of phenotypes compared to the parent code that appears before the decimal. In our case study, the data we make use of include mostly upper hierarchy phenotypes. More detailed hierarchical phenotypes are for the most part absent from our network. Users should be careful about including extensive hierarchical structure in their input data when generating DDNs through NETMAGE. Including phenotypes that are essentially identical to one another will introduce unnecessary nodes and edges in the network, in the process clouding more significant disease connections.

In terms of future work for this case study, it would be interesting to compare the edges in our DDN with known disease comorbidities. We can take disease occurrence data from an external electronic health record and evaluate phi correlations between all pairs of phenotypes. Comparison of these co-occurrences to the genetic associations in our PheWAS may give us an indication if the DDN is a reasonable representation of disease connections.

5. Runtime Analysis

As a test of runtime for NETMAGE, we constructed DDNs from random subsets of the PheWAS data used to create the UKBB DDN and determined the time it took for each network to be generated. Five networks were each generated from collections of 50, 100, 250, 500, and 1000 phenotypes. These DDNs were constructed in both the Fruchterman-Reingold and Force Atlas 2

layouts from Gephi[13], resulting in a total of 50 graphs for runtime analysis. The average time to create a network seems to grow in O(n^2) as the number of phenotypes increases (Table 3). This behavior makes sense, as runtime depends on not only the number of phenotypes included in the input data, but also the number of variants being tested. Indeed, if each additional phenotype added to the network will have multiple associated variants, then the inclusion of nodes will tend to exponentially increase the number of edges assuming a low clustering coefficient in the network.

Table 3. A table of run times (in seconds) for DDN generation given input datasets with different numbers of phenotypes. These times measure how long it takes for the server to generate the network after the "submit" button has been clicked – in all instances, files have already been uploaded to the server. Upload speeds for files will vary depending on user bandwidth. Five different datasets were constructed for each count of phenotypes to evaluate runtime, and the mean and standard deviation of time for the five runs is also provided for each row. Finally, runtime for the full input UKBB case study is included in the last row of the table.

Phenotype Count	**Server runtime (in seconds) to generate network after receiving HTTP request**													
	Fruchterman-Reingold Layout							***Force Atlas 2 Layout***						
	1	***2***	***3***	***4***	***5***	***Mean***	***SD***	***1***	***2***	***3***	***4***	***5***	***Mean***	***SD***
50	3.07	2.34	2.86	2.31	2.76	2.67	0.33	2.46	2.48	2.93	2.43	3.00	2.66	0.28
100	3.26	3.49	4.29	3.61	3.52	3.63	0.39	3.43	4.14	4.37	4.62	3.58	4.03	0.51
250	6.60	5.20	6.77	6.62	5.56	6.15	0.72	6.74	5.31	6.36	6.92	5.90	6.25	0.65
500	11.21	11.85	12.53	10.94	9.91	11.29	0.99	11.68	12.04	12.49	11.21	9.33	11.35	1.22
1000	28.27	28.77	30.19	27.01	29.52	28.75	1.22	29.37	28.35	29.84	27.23	30.23	29.00	1.22
UKBB DDN	48.60					N/A	N/A	39.43					N/A	N/A

6. Discussion and Conclusions

NETMAGE is a toolkit for the network-based interactive visualization of PheWAS summary data. The goal of this software is to improve the ease of visualization of genetic associations across diseases and to facilitate large-scale genetic analysis of the human diseasome. While the UKBB data used for our case study consisted of entirely binary phenotypes, NETMAGE is also applicable to quantitative traits. Indeed, in such a situation, the continuous value of the quantitative phenotype, such as a laboratory test measure like A1C level, is used as the outcome variable in the PheWAS. This process provides a more detailed degree of association between the severity of the trait and genetic variants, as compared to the identification of associations between a presence or absence of the trait with variants.

A key point to note regarding NETMAGE is that the output DDNs will provide only as much information as the input data. Indeed, NETMAGE is an exploratory tool intended to help visualize connections between diseases. Including summary PheWAS data that provides insight into the statistical associations between phenotypes will yield an associative map but will tell us nothing about causality. Associations identified through PheWAS are often spurious, so any sort of analyses performed on these data must take this information into consideration. Nevertheless, these kinds of associative visualizations are still useful for the study of disease and may help identify connections between phenotypes and genetic variants, generate new hypotheses, and suggest future experiments that can be conducted. For a visualization that gives stronger insight into the causal connections between traits, one could potentially input the results of a Mendelian Randomization experiment.

Several future directions exist for NETMAGE. First is the inclusion of directionality in the network – as of now, DDNs produced by NETMAGE give no indication regarding the direction of association between phenotypes. Using beta values for the association between phenotypes and genetic variants would be a useful inclusion, aiding in clinical interpretation of the network. We will also allow for the concurrent selection of multiple nodes within the DDN. The current NETMAGE user interface allows only one node to be selected at a time. The ability to select multiple nodes will allow clinicians to quickly identify if two phenotypes are associated in the network. We also hope to enhance NETMAGE to allow for the construction of gene-based DDNs from variant-based data by including variant-to-gene mapping as a part of the website. Finally, we will allow users to create variant-variant networks instead of disease-disease networks, which depict the connections between genetic variants (for instance, SNPs) based upon associations with phenotypes.

Ultimately, NETMAGE will give researchers and clinicians insight into the underlying genetic architecture of disease complications. The impact of our work will be a tool that allows for the potential identification of new gene targets that can be investigated in follow-up studies of pleiotropy and drug discovery. We hope that this software will contribute to new potential discoveries in personalized medicine and that it helps facilitate the advancement of network medicine studies into the genetics of disease co-occurrences.

Availability of supporting source code and requirements

- Project name: NETMAGE
- Project home page: https://hdpm.biomedinfolab.com/netmage/
- Source code: https://github.com/dokyoonkimlab/netmage
- Operating system(s): Platform independent
- Programming language: Python, HTML, JavaScript
- Other requirements: None
- Contact: dokyoon.kim@pennmedicine.upenn.edu

Data Availability

Supporting data and materials are available in the *GigaDB* database[28].

Funding

This work has been supported by the NIGMS R01 GM138597 and S10OD023495.

Conflict of Interest: none declared.

Citations

1. Valderas JM, Starfield B, Sibbald B, Salisbury C, Roland M. Defining Comorbidity: Implications for Understanding Health and Health Services. *The Annals of Family Medicine*. 2009;7(4):357-363. doi:10.1370/afm.983

2. Bush WS, Oetjens MT, Crawford DC. Unravelling the human genome–phenome relationship using phenome-wide association studies. *Nat Rev Genet*. 2016;17(3):129-145. doi:10.1038/nrg.2015.36

3. Rubio-Perez C, Guney E, Aguilar D, et al. Genetic and functional characterization of disease associations explains comorbidity. *Sci Rep*. 2017;7(1):6207. doi:10.1038/s41598-017-04939-4

4. Denny J, Bastarache L, Roden D. Systematic comparison of phenome-wide association study of electronic medical record data and genome-wide association study data. *Nature Biotechnology*. 2013;31:1102-1111. doi:10.1038/nbt.2749

5. Barabási AL, Gulbahce N, Loscalzo J. Network medicine: a network-based approach to human disease. *Nat Rev Genet*. 2011;12(1):56-68. doi:10.1038/nrg2918

6. Goh KI, Cusick ME, Valle D, Childs B, Vidal M, Barabasi AL. The human disease network. *Proceedings of the National Academy of Sciences*. 2007;104(21):8685-8690. doi:10.1073/pnas.0701361104

7. Verma A, Bang L, Miller J, et al. Human-Disease Phenotype Map Derived from PheWAS across 38,682 Individuals. *AJHG*. 2019;104(1):55-64. doi:10.1016/j.ajhg.2018.11.006

8. Gao XR, Huang H. PleioNet: a web-based visualization tool for exploring pleiotropy across complex traits. *Bioinformatics*. 2019;35(20):4179-4180. doi:10.1093/bioinformatics/btz179

9. Kortemeier E, Ramos P, Hunt K, Kim H, Hardiman G, Chung D. ShinyGPA: An interactive visualization toolkit for investigating pleiotropic architecture using GWAS datasets. *PLOS ONE*. 2018;13(1). doi:10.1371/journal.pone.0190949

10. George G, Gan S, Huang Y, et al. PheGWAS: a new dimension to visualize GWAS across multiple phenotypes. *Bioinformatics*. 2020;36(8):2500-2505. doi:10.1093/bioinformatics/btz944

11. Gagliano Taliun SA, VandeHaar P, Boughton AP, et al. Exploring and visualizing large-scale genetic associations by using PheWeb. *Nat Genet*. 2020;52(6):550-552. doi:10.1038/s41588-020-0622-5

12. Strayer N, Shirey-Rice J, Shyr Y, Denny J, Pulley J, Xu Y. PheWAS-ME: A web-app for interactive exploration of multimorbidity patterns in PheWAS. *medRXiv*. Published online June 2, 2020. doi:10.1101/19009480

13. Bastian M, Heymann S, Jacomy M. Gephi: An Open Source Software for Exploring and Manipulating Networks. In: Association for the Advancement of Artificial Intelligence; 2009.

14. Oxford Internet Institue. Interactive Visualizations. Published 2020. http://blogs.oii.ox.ac.uk/vis/

15. Jacomy A, Plique G. *Sigmajs*. http://sigmajs.org

16. NETMAGE website https://hdpm.biomedinfolab.com/netmage/

17. Sherry ST. dbSNP: the NCBI database of genetic variation. *Nucleic Acids Research*. 2001;29(1):308-311. doi:10.1093/nar/29.1.308

18. Zhou W, Nielsen J, Fritsche L, et al. Efficiently controlling for case-control imbalance and sample relatedness in large-scale genetic association studies. *Nature Genetics*. 2018;50:1335-1341. doi:10.1038/s41588-018-0184-y

19. *UK Biobank*. https://www.ukbiobank.ac.uk

20. UK BioBank Disease-Disease Network map https://hdpm.biomedinfolab.com/ddn/ukbb

21. Wei WQ, Bastarache LA, Carroll RJ, et al. Evaluating phecodes, clinical classification software, and ICD-9-CM codes for phenome-wide association studies in the electronic health record. Rzhetsky A, ed. *PLoS ONE*. 2017;12(7):e0175508. doi:10.1371/journal.pone.0175508

22. Altshuler D, Daly MJ, Lander ES. Genetic Mapping in Human Disease. *Science*. 2008;322(5903):881-888. doi:10.1126/science.1156409

23. Purcell S, Neale B, Todd-Brown K, et al. PLINK: A Tool Set for Whole-Genome Association and Population-Based Linkage Analyses. *The American Journal of Human Genetics*. 2007;81(3):559-575. doi:10.1086/519795

24. Guo M, Yu Y, Wen T, et al. Analysis of disease comorbidity patterns in a large-scale China population. *BMC Med Genomics*. 2019;12(S12):177. doi:10.1186/s12920-019-0629-x

25. Mitsutake S, Ishizaki T, Teramoto C, Shimizu S, Ito H. Patterns of Co-Occurrence of Chronic Disease Among Older Adults in Tokyo, Japan. *Prev Chronic Dis*. 2019;16:180170. doi:10.5888/pcd16.180170

26. Zhou W, Brumpton B, Asvold B. GWAS of thyroid stimulating hormone highlights pleiotropic effects and inverse association with thyroid cancer. *Nature Communications*. 2020;11. doi:10.1038/s41467-020-17718-z

27. Bi M, Kao WH, Boerwinkle E, et al. Association of rs780094 in GCKR with Metabolic Traits and Incident Diabetes and Cardiovascular Disease: The ARIC Study. *PLOS ONE*. 2010;5. doi:10.1371/journal.pone.0011690

28. Sriram V; Shivakumar M; Jung S; Nam Y; Bang L; Verma A; Lee S; Choe EK; Kim D. Supporting data for "NETMAGE: A Human Disease Phenotype Map Generator for the Network-based Visualization of PheWAS Results" *GigaScience* Database. 2022. http://dx.doi.org/10.5524/100975

Supplementary Data

Table S1. A table of phenotypes with the highest centrality measures in the UKBB DDN. Diseases marked in bold appear multiple times as the most central nodes based upon our different network measures.

Phenotype	***PheCode***	***Attribute***	***Value***
**Hypothyroidism NOS**	244.4	Degree	83
**Disorders of lipoid metabolism**	272	Degree	79
**Type 2 diabetes**	250.2	Degree	79
Diabetes mellitus	250	Degree	77
**Hyperlipidemia**	272.1	Degree	76
**Celiac disease**	557.1	Weighted Degree	$1.27*10^5$
Non-celiac intestinal malabsorption	557	Weighted Degree	$1.26*10^5$
**Hypothyroidism NOS**	244.4	Weighted Degree	$7.48*10^4$
**Hypothyroidism**	244	Weighted Degree	$7.39*10^4$
**Type 1 diabetes**	250.1	Weighted Degree	$6.53*10^4$
**Psoriasis**	696	Weighted Degree	$5.09*10^4$
Psoriasis NOS	696.4	Weighted Degree	$5.11*10^4$
Disorders of muscle, ligament, and fascia	728	Closeness Centrality	1.00
Fasciitis	728.7	Closeness Centrality	1.00
Other retinal disorders	362	Closeness Centrality	1.00
Skin cancer	172	Betweenness Centrality	$2.15*10^3$
**Disorders of lipoid metabolism**	272	Betweenness Centrality	$1.97*10^3$
**Hyperlipidemia**	272.1	Betweenness Centrality	$1.97*10^3$
Essential hypertension	401.1	Betweenness Centrality	$1.84*10^3$
Hypertension	401	Betweenness Centrality	$1.19*10^3$
Coronary atherosclerosis	411.4	Betweenness Centrality	$7.72*10^2$
Intestinal malabsorption	557	Eigenvector Centrality	1.00
**Celiac disease**	557.1	Eigenvector Centrality	1.00
**Hypothyroidism NOS**	244.4	Eigenvector Centrality	0.98
**Hypothyroidism**	244	Eigenvector Centrality	0.98
**Type 1 diabetes**	250.1	Eigenvector Centrality	0.95

**Type 2 diabetes**	250.2	Eigenvector Centrality	0.93
Rheumatoid arthritis	714.1	Eigenvector Centrality	0.89
Other inflammatory polyarthropathies	714	Eigenvector Centrality	0.89
**Psoriasis**	696	Eigenvector Centrality	0.86
