# Supplementary material for: NETMAGE: A human disease phenotype map generator for the network-based visualization of phenome-wide association study results
Source: Gigascience. 2022 Feb 15;11:giac002. doi: 10.1093/gigascience/giac002 (PMC8848314; doi:10.1093/gigascience/giac002)
Supplement: giac002_Supplemental_File [file giac002_supplemental_file.pdf]

Supplementary Data

Table S1. A table of phenotypes with the highest centrality measures in the UKBB DDN. Diseases marked in bold appear multiple times as the most central nodes based upon our different network measures.

Phenotype	***PheCode***	***Attribute***	***Value***
**Hypothyroidism NOS**	244.4	Degree	83
**Disorders of lipoid metabolism**	272	Degree	79
**Type 2 diabetes**	250.2	Degree	79
Diabetes mellitus	250	Degree	77
**Hyperlipidemia**	272.1	Degree	76
**Celiac disease**	557.1	Weighted Degree	$1.27*10^5$
Non-celiac intestinal malabsorption	557	Weighted Degree	$1.26*10^5$
**Hypothyroidism NOS**	244.4	Weighted Degree	$7.48*10^4$
**Hypothyroidism**	244	Weighted Degree	$7.39*10^4$
**Type 1 diabetes**	250.1	Weighted Degree	$6.53*10^4$
**Psoriasis**	696	Weighted Degree	$5.09*10^4$
Psoriasis NOS	696.4	Weighted Degree	$5.11*10^4$
Disorders of muscle, ligament, and fascia	728	Closeness Centrality	1.00
Fasciitis	728.7	Closeness Centrality	1.00
Other retinal disorders	362	Closeness Centrality	1.00
Skin cancer	172	Betweenness Centrality	$2.15*10^3$
**Disorders of lipoid metabolism**	272	Betweenness Centrality	$1.97*10^3$
**Hyperlipidemia**	272.1	Betweenness Centrality	$1.97*10^3$
Essential hypertension	401.1	Betweenness Centrality	$1.84*10^3$
Hypertension	401	Betweenness Centrality	$1.19*10^3$
Coronary atherosclerosis	411.4	Betweenness Centrality	$7.72*10^2$
Intestinal malabsorption	557	Eigenvector Centrality	1.00
**Celiac disease**	557.1	Eigenvector Centrality	1.00
**Hypothyroidism NOS**	244.4	Eigenvector Centrality	0.98
**Hypothyroidism**	244	Eigenvector Centrality	0.98
**Type 1 diabetes**	250.1	Eigenvector Centrality	0.95

**Type 2 diabetes**	250.2	Eigenvector Centrality	0.93
Rheumatoid arthritis	714.1	Eigenvector Centrality	0.89
Other inflammatory polyarthropathies	714	Eigenvector Centrality	0.89
**Psoriasis**	696	Eigenvector Centrality	0.86
