# Supplementary material for: NETMAGE: A human disease phenotype map generator for the network-based visualization of phenome-wide association study results
Source: Gigascience. 2022 Feb 15;11:giac002. doi: 10.1093/gigascience/giac002 (PMC8848314; doi:10.1093/gigascience/giac002)
Supplement: giac002_Reviewer_1_Report_Original_Submission — Sarah Gagliano Taliun, PhD -- 8/3/2021 Reviewed [file giac002_reviewer_1_report_original_submission.pdf]

Reviewer Report

Title: NETMAGE: A Human Disease Phenotype Map Generator for the Network-based Visualization of PheWAS Results

Version: Original Submission **Date:** 8/3/2021

Reviewer name: Sarah Gagliano Taliun, PhD

Reviewer Comments to Author:

Sriram et al. introduce an open-source web-based tool NETMAGE to produce interactive disease-disease network (DDN) visualizations of biobank-level phenome-wide association summary statistics. The concept is interesting and relevant, but my major concern is regarding the interpretability of the DDN for researchers and clinicians to draw insights intuitively.

Comments on the manuscript:

Generally well written and logical flow. Some minor errors (e.g. "an SNP" rather than "a SNP") and some headers could be improved for readability (e.g. "Testing" is vague; this section really only touches upon Run time).

Figure 1- Displaying a single Manhattan plot for "PheWAS Summary Statistics" is not very intuitive. It makes me think of a single GWAS rather than a phenome-wide set of GWAS run on a Biobank. Perhaps revise the image.

Is the disease-disease network only applicable to case/control studies? Could there be an extension to quantitative traits, and if so, would that be pertinent for discoveries?

The authors refer to "SNPs" throughout to define genetic variation. If the summary statistics contains another type of variation (e.g. indels), are those associations still used? If so, I would suggest using a more generic term to define the genetic variation.

The discussion seems underdeveloped. Discussion of limitations rather than only future work would be helpful.

Case study-- The authors could improve the interpretability/discussion of the UKB PheWAS example. This is one of my largest concerns because the author state that the tool can help researchers and clinicians get insight into the underlying genetic architecture of disease complications; however, the case study part of the manuscript is quite technical and could be challenging to interpret for someone without network experience; e.g. Table 2.

Additionally, more details should be provided on the underlying summary statistics used (e.g. some details can be found on the About page of the HRC-imputed UKB PheWeb page: https://pheweb.org/UKB-SAIGE/about).

The authors list additional filtering that they performed on the summary statistics, but it appears that some details are missing. For instance, how many traits remain after the case count filtering is applied? Also, what is used as a reference for the LD-pruning in PLINK?

Run time-- I am wondering why Table 3 (run time for subsets of the UKBB data) ends at 1000 phenotypes. It would be interesting to see the run time that is close to case example (e.g. possibly adding a column for the total number of phenotypes used in the UKBB DDN). Additionally, this section

gives the impression that run time only depend on the number of phenotypes? I would assume that run time should also depend on the number of variants that were tested.
Comments on the online tool:
It is nice that on each page the authors have allowed users to download a pdf of the image and also the data behind the image (e.g. edge-map, node-map, etc.). The zoom-in feature for the visualization is also useful, as is the short video tutorial.
I think that the search bar would be more user-friendly if suggestions automatically came up when the user begins to type. Additionally, displaying the list of "associated SNPs" in a (sortable and/or searchable) table (with some annotations, such as chr, position, closest gene, consequence, rather than just rsID) could be a neater and more informative way to show these data, rather than simply as it appears currently as a list in the "information pane".
My comment on interpretability for researchers and clinicians comes up again: I am not sure how useful/interpretable some of the search categories are for users to intuitively draw insights; for instance, number of triangles, page range, etc. I think the authors should really focus on the intuitiveness for the target audience so that the tool can have more impact.

Level of Interest

Please indicate how interesting you found the manuscript: Choose an item.

Quality of Written English

Please indicate the quality of language in the manuscript: Choose an item.

Declaration of Competing Interests

Please complete a declaration of competing interests, considering the following questions:

- Have you in the past five years received reimbursements, fees, funding, or salary from an organisation that may in any way gain or lose financially from the publication of this manuscript, either now or in the future?
- Do you hold any stocks or shares in an organisation that may in any way gain or lose financially from the publication of this manuscript, either now or in the future?
- Do you hold or are you currently applying for any patents relating to the content of the manuscript?
- Have you received reimbursements, fees, funding, or salary from an organization that holds or has applied for patents relating to the content of the manuscript?
- Do you have any other financial competing interests?
- Do you have any non-financial competing interests in relation to this paper?

If you can answer no to all of the above, write 'I declare that I have no competing interests' below. If your reply is yes to any, please give details below.

I declare that I have no competing interests

I agree to the open peer review policy of the journal. I understand that my name will be included on my report to the authors and, if the manuscript is accepted for publication, my named report including any attachments I upload will be posted on the website along with the authors' responses. I agree for my report to be made available under an Open Access Creative Commons CC-BY license (http://creativecommons.org/licenses/by/4.0/). I understand that any comments which I do not wish to be included in my named report can be included as confidential comments to the editors, which will not be published.

Choose an item.

To further support our reviewers, we have joined with Publons, where you can gain additional credit to further highlight your hard work (see: https://publons.com/journal/530/gigascience). On publication of this paper, your review will be automatically added to Publons, you can then choose whether or not to claim your Publons credit. I understand this statement.

Yes Choose an item.
