# Supplementary material for: NETMAGE: A human disease phenotype map generator for the network-based visualization of phenome-wide association study results
Source: Gigascience. 2022 Feb 15;11:giac002. doi: 10.1093/gigascience/giac002 (PMC8848314; doi:10.1093/gigascience/giac002)
Supplement: giac002_Reviewer_2_Report_Original_Submission — Dongjun Chung -- 8/15/2021 Reviewed [file giac002_reviewer_2_report_original_submission.pdf]

Reviewer Report

Title: NETMAGE: A Human Disease Phenotype Map Generator for the Network-based Visualization of PheWAS Results

Version: Original Submission **Date:** 8/15/2021

Reviewer name: Dongjun Chung

Reviewer Comments to Author:

In this paper, the authors developed the humaN disease phenotype Map Generator (NETMAGE), a web-based tool that produces interactive disease-disease network visualization based on PhEWAS summary statistics. The tool proposed in this manuscript has important implication and utility for biological and clinical studies. The manuscript is also overall well-written and clearly described NETMAGE. However, there are still some aspects I hope the authors to address. I provide my comments in detail below.
Major comments:
1. I tried the web interface Human-Disease Phenotype Map (https://hdpm.biomedinfolab.com), which utilizes NETMAGE. I found that sometimes it takes some time for the network to appear. While the network is loaded, only the gray empty space with the side panel is shown. I recommend the authors to show the progress bar while loading the network, especially when it is first loaded, to avoid users to think that their web browser is frozen.
2. In the Search bar, it is not always trivial to guess what to enter, especially for Phenotype Name, Associated SNPs, and category. Auto-completion features for these variables will significantly facilitate users' convenience.
3. Meaning of edges is somewhat unclear to me. Are the existence and the weights of edges purely based on the number of shared SNPs or are they based on any statistical methods?
4. When the weights of edges are calculated, are the marginal counts taken into account? The same number of shared SNPs can have different meanings when the disease to which this edge is connected has a small number of associated SNPs vs. a large number of associated SNPs. How is this factor considered?
5. The network generated by the Human-Disease Phenotype Map (https://hdpm.biomedinfolab.com) is usually huge and complex with a large number of edges. As a result, it is often not straightforward to understand the generated network. This is partially relevant to the fact that the network layout is static, i.e., locations of nodes remain the same regardless of which subnetworks are chosen. If the network layout is optimized for each subnetwork, it should be much easier for users to understand the network architecture. Given this, I recommend the authors to consider updating the network layout interactively when a subnetwork is selected.
6. When a subnetwork is chosen, the "Information Pane" appears. In this pane, it might be helpful for users if the authors provide some quick help link for each network score, e.g., how to interpret PageRank scores, etc.
7. In the "Information Pane", a long list of SNPs is provided for "Associated SNPs" but it is not easy to use this list. I recommend the authors to make it downloadable as a table so that users can do downstream

analysis. In addition, it will significantly facilitate users' convenience if each SNP ID is chosen, it brings the user to the relevant database, e.g., dbSNP. In this way, users can easily check where it is located in the sense of chromosome, gene, exon/intron/promoter/intergenic, etc. Alternatively, the authors can consider to use a quick information table (SNP ID, gene name, exon/intron/promoter/intergenic) instead of simply providing as a list.

Level of Interest

Please indicate how interesting you found the manuscript: Choose an item.

Quality of Written English

Please indicate the quality of language in the manuscript: Choose an item.

Declaration of Competing Interests

Please complete a declaration of competing interests, considering the following questions:

- Have you in the past five years received reimbursements, fees, funding, or salary from an organisation that may in any way gain or lose financially from the publication of this manuscript, either now or in the future?
- Do you hold any stocks or shares in an organisation that may in any way gain or lose financially from the publication of this manuscript, either now or in the future?
- Do you hold or are you currently applying for any patents relating to the content of the manuscript?
- Have you received reimbursements, fees, funding, or salary from an organization that holds or has applied for patents relating to the content of the manuscript?
- Do you have any other financial competing interests?
- Do you have any non-financial competing interests in relation to this paper?

If you can answer no to all of the above, write 'I declare that I have no competing interests' below. If your reply is yes to any, please give details below.

I declare that I have no competing interests

I agree to the open peer review policy of the journal. I understand that my name will be included on my report to the authors and, if the manuscript is accepted for publication, my named report including any attachments I upload will be posted on the website along with the authors' responses. I agree for my report to be made available under an Open Access Creative Commons CC-BY license (http://creativecommons.org/licenses/by/4.0/). I understand that any comments which I do not wish to be included in my named report can be included as confidential comments to the editors, which will not be published.

Choose an item.

To further support our reviewers, we have joined with Publons, where you can gain additional credit to further highlight your hard work (see: https://publons.com/journal/530/gigascience). On publication of this paper, your review will be automatically added to Publons, you can then choose whether or not to claim your Publons credit. I understand this statement.

Yes Choose an item.
