# Supplementary material for: NETMAGE: A human disease phenotype map generator for the network-based visualization of phenome-wide association study results
Source: Gigascience. 2022 Feb 15;11:giac002. doi: 10.1093/gigascience/giac002 (PMC8848314; doi:10.1093/gigascience/giac002)
Supplement: giac002_Reviewer_3_Report_Original_Submission — Yaomin Xu -- 9/1/2021 Reviewed [file giac002_reviewer_3_report_original_submission.pdf]

Reviewer Report

Title: NETMAGE: A Human Disease Phenotype Map Generator for the Network-based Visualization of PheWAS Results

Version: Original Submission **Date:** 9/1/2021

Reviewer name: Yaomin Xu

Reviewer Comments to Author:

The authors presented a web tool - NETMAGE that produces an interactive network-based visualization of disease cross-phenotype relationships based on PheWAS summary statistics. NETMAGE provides search functions for various attributes and selecting nodes to view related phenotypes, associated SNPs, and various network statistics. As a use case, authors used NETMAGE to construct a network from UK BioBank (UKBB) PheWAS summary statistic data. The purpose of the tool as claimed by the authors is to provide a holistic, network-based view for an intuitive understanding of the relationships between disease phenotypes and to help analyze the shared genetic etiology.

Major comments:

A DDN based on true genetic associations is useful for understanding complex disease comorbidities and their shared genetic etiology (pleiotropy). An interactive web tool to explore such a complex networked information could be highly useful for the proposed purposes of this tool. However, the EHR/Biobank PheWAS associations data are statistical in nature and commonly with small effect sizes. The reported genetic associations often are not well understood at the mechanistic level, and many genetic associations are spurious. Although certain positive findings can be observed from the disease network generated by NETMAGE, it's of concern the general usability of the current implementation of the tool in order to facilitate novel applications in drug design and personalized medicine, which requires the genetic associations to best represent the underlying true causal mechanism. Further work is needed to verify the genetic associations reported from PheWAS to minimize the impact of spurious associations.

Network edges based on SNPs without considering the linkage disequilibrium (LD) between SNPs is misleading and could miss a significant portion of associations that should be linked between diseases if the LD correlations are considered. When construct the network using NETMAGE, the LD correlation between SNPs should be considered.

For the reported DDN and its statistics to be relevant to true disease - disease relationships, the quality of disease diagnosis using Phecode should be considered. Phecodes are based on ICD codes that are known to be noisy. The accuracy of ICD can be as low as only 50%. Ignoring this limitation and treating disease diagnoses from Phecodes as gold standards or as precise and accurate may result in irrelevant and misleading findings.

Phecodes are hierarchical. For example, parent codes are three digits (008), and each additional digit after decimal point indicates a subset of ICD codes of the parent code (008.5 and 008.52). So here a code 008.52 implies 008.5 also 008. What's the impact of this hierarchy to the NETMAGE network and the inferences to be made based on the network?

Minor comments:

On Page 9, you said "Out of the 2189 edges for which phi correlations could be calculated, 1811 (82.73%) appeared in the DDN. This behavior suggests that our genetic associations identified by our PheWAS results serve as a reasonable approximation of disease co-occurrences".
This is expected because both phi correlation and PheWAS analyses were performed on the same dataset. If a pair of disease highly co-occur in the dataset, you would expect a strong correlation on their genetic associations analyzed on the same dataset. However, it may not be generalizable that the genetic associations from PheWAS are a reasonable approximation to disease co-occurrences.
The disease-SNP relationships from the PheWAS analysis result are bipartite. Even though NETMAGE focuses on the projected disease-disease network, the information about how specific SNPs link to their corresponding disease pairs is important. For example, in your UKBB-based network (https://hdpm.biomedinfolab.com/ddn/ukbb), when a specific disease is selected, a subgraph of the selected disease and other disease linked to the selected one are showing, but sonly a lump of SNPs without linking to their specific disease pair is provided. This is not helpful. Also annotating those SNPs their genetic context could be very useful for users to quickly grasp the nature of the genetic associations in the subgraph.

Level of Interest

Please indicate how interesting you found the manuscript: Choose an item.

Quality of Written English

Please indicate the quality of language in the manuscript: Choose an item.

Declaration of Competing Interests

Please complete a declaration of competing interests, considering the following questions:

- Have you in the past five years received reimbursements, fees, funding, or salary from an organisation that may in any way gain or lose financially from the publication of this manuscript, either now or in the future?
- Do you hold any stocks or shares in an organisation that may in any way gain or lose financially from the publication of this manuscript, either now or in the future?
- Do you hold or are you currently applying for any patents relating to the content of the manuscript?
- Have you received reimbursements, fees, funding, or salary from an organization that holds or has applied for patents relating to the content of the manuscript?
- Do you have any other financial competing interests?
- Do you have any non-financial competing interests in relation to this paper?

If you can answer no to all of the above, write 'I declare that I have no competing interests' below. If your reply is yes to any, please give details below.

I declare that I have no competing interests. The authors cited our paper.

I agree to the open peer review policy of the journal. I understand that my name will be included on my report to the authors and, if the manuscript is accepted for publication, my named report including any attachments I upload will be posted on the website along with the authors' responses. I agree for my report to be made available under an Open Access Creative Commons CC-BY license (http://creativecommons.org/licenses/by/4.0/). I understand that any comments which I do not wish to be included in my named report can be included as confidential comments to the editors, which will not be published.

Choose an item.

To further support our reviewers, we have joined with Publons, where you can gain additional credit to further highlight your hard work (see: https://publons.com/journal/530/gigascience). On publication of this paper, your review will be automatically added to Publons, you can then choose whether or not to claim your Publons credit. I understand this statement.

Yes Choose an item.
